# Self-Assembled Block Copolymers as a Facile Pathway to Create Functional Nanobiosensor and Nanobiomaterial Surfaces

**DOI:** 10.3390/polym16091267

**Published:** 2024-05-01

**Authors:** Marion Ryan C. Sytu, David H. Cho, Jong-in Hahm

**Affiliations:** 1 Department of Chemistry, Georgetown University, 37th & O Sts. NW., Washington, DC 20057, USA; 2 National Institute of Biomedical Imaging and Bioengineering, National Institutes of Health, 9000 Rockville Pike, Bethesda, MD 20892, USA; dc1210@georgetown.edu

**Keywords:** BCP nanobiotechnology, BCP self-assembly, BCP thin films, self-assembled BCP nanopatterns, protein arrays, nanobiosensors, biomaterials, proteins, cells

## Abstract

Block copolymer (BCP) surfaces permit an exquisite level of nanoscale control in biomolecular assemblies solely based on self-assembly. Owing to this, BCP-based biomolecular assembly represents a much-needed, new paradigm for creating nanobiosensors and nanobiomaterials without the need for costly and time-consuming fabrication steps. Research endeavors in the BCP nanobiotechnology field have led to stimulating results that can promote our current understanding of biomolecular interactions at a solid interface to the never-explored size regimes comparable to individual biomolecules. Encouraging research outcomes have also been reported for the stability and activity of biomolecules bound on BCP thin film surfaces. A wide range of single and multicomponent biomolecules and BCP systems has been assessed to substantiate the potential utility in practical applications as next-generation nanobiosensors, nanobiodevices, and biomaterials. To this end, this Review highlights pioneering research efforts made in the BCP nanobiotechnology area. The discussions will be focused on those works particularly pertaining to nanoscale surface assembly of functional biomolecules, biomolecular interaction properties unique to nanoscale polymer interfaces, functionality of nanoscale surface-bound biomolecules, and specific examples in biosensing. Systems involving the incorporation of biomolecules as one of the blocks in BCPs, i.e., DNA–BCP hybrids, protein–BCP conjugates, and isolated BCP micelles of bioligand carriers used in drug delivery, are outside of the scope of this Review. Looking ahead, there awaits plenty of exciting research opportunities to advance the research field of BCP nanobiotechnology by capitalizing on the fundamental groundwork laid so far for the biomolecular interactions on BCP surfaces. In order to better guide the path forward, key fundamental questions yet to be addressed by the field are identified. In addition, future research directions of BCP nanobiotechnology are contemplated in the concluding section of this Review.

## 1. Introduction

The development of functional biosensors has long drawn considerable research interests across many different disciplines in fundamental science, biotechnology, and medicine [1,2,3,4,5,6,7,8,9,10,11]. One of the notable trends in recent efforts for biosensor development involves high miniaturization [6,8,12,13,14] and flexibility/wearability [3,5,7,9,15,16]. Advances in nanoscience continuously propel such a drive to create flexible and miniaturized biosensors, permitting high-throughput detection of bioanalytes that are held on an array of nanometer-sized sensor surfaces in a flexible setting. The majority of conventional biosensors are fabricated by top-down approaches such as photolithography, soft (microcontact printing) lithography, and inkjet printing, which can be costly and time-consuming [2,13,17,18,19,20,21,22,23,24,25]. Fabrication techniques relying on conventional lithographic procedures also present limitations in the size of the smallest possible sensor unit that can be individually addressed. This is due to the optical diffraction limit of light-based lithographic tools commonly used in the fabrication process. Although there exist lithographic tools of higher spatial resolution such as electron-beam lithography [26,27,28], scanning probe-based lithography [29,30] and nanoimprint lithography [31,32], the involvement of these procedures can lead to an even slower fabrication process and a higher production cost. Hence, alternative approaches based on self-assembly have emerged to create nanoscopic patterns of individually addressable biosensor surfaces and nanoscale bioreactors in simple steps [17,33,34,35,36,37,38,39].

The remarkable self-assembly behaviors of block copolymers (BCPs) have been well-recognized as one of the most versatile and convenient mechanisms to exploit a bottom-up assembly approach in organizing nanoscale features [40,41,42,43,44,45,46,47,48]. BCPs can be synthesized from a rich selection of monomers, whose chemical compositions can be tuned to match the desired functionalities for their applications [43,49,50,51,52,53,54]. It is also straightforward to create BCPs into thin structures that can be flexible and wearable. Furthermore, there exists a wealth of theoretical, computational, and experimental works performed to understand the phase separation behaviors of BCPs [40,45,46,47,49,55,56,57,58,59,60,61,62,63,64,65]. Owing to these efforts, nanoscale features resulting from BCPs’ phase separation processes and their two-/three-dimensional (2D/3D) periodicities have been well-characterized. The size and shape of these nanopatterns that can be controlled thermodynamically and kinetically have also been mapped out for many BCPs. As such, nanoscale BCP surface patterns have extensively been utilized as templates to organize inorganic nanomaterials in BCP lithography [33,34,42,49,66,67,68,69,70,71,72,73,74]. The first attempt to use BCP nanopatterns for assembling biomolecules such as proteins was undertaken in mid 2000s [75]. Many ensuing endeavors have since been made in the field of BCP nanobiotechnology as represented in Table 1. The various BCP–biomolecule systems in Table 1 summarize the stimulating research endeavors and findings that will be discussed in this Review. All these efforts have successfully demonstrated the application of underlying BCP nanopatterns in controlling the spatial density, large area assembly, adsorption/desorption dynamics, biofunctionality, and other important interfacial characteristics of biologically relevant molecules such as proteins, peptides, biomineral nanocrystals, cell adhesive molecules, and cells. The focus of this Review is to provide a comprehensive and detailed overview of those research efforts pertaining to nanoscale surface assembly of functional biomolecules, biomolecular interaction properties at nanoscale polymer interfaces, functionality of nanoscale surface-bound biomolecules, as well as specific examples in biosensing.

## 2. Block Copolymers as Nanoscale Templates

### 2.1. Block Copolymer Nanostructures in Bulk

BCPs are synthesized by covalently linking two or more, chemically distinct, polymer blocks via methods such as atom transfer free radical polymerization (ATRP) and reversible addition fragmentation chain transfer (RAFT) [43,49,50,51,52,53,54]. In bulk, BCPs self-assemble into various nanostructures with a tunable periodicity typically in the range of 5–100 nm through a process known as microphase separation. The phase separation processes of BCPs occur as a direct consequence of self-assembly driven by chemically incompatible polymer segments in a given BCP to maximize (minimize) the spatial contact between similar (dissimilar) blocks. However, these forces driving phase separation are countered by the entropic forces of polymer chain mixing since the different blocks of the BCP are covalently bonded together. The BCP microphase separation is ultimately achieved by a balance between forces associated with separating and mixing and hence, the process is thermodynamically driven by enthalpic and entropic parameters. The enthalpic term of the process is defined by the Flory–Huggins interaction parameter (χ) which is related to the free energy cost between the different polymer blocks. The interaction parameter of χ is inversely proportional to temperature (T). The entropic term of mixing is affected by the degree of polymerization (N) and the relative composition fraction of polymer blocks in terms of volume fraction (f).

**Figure 1:**
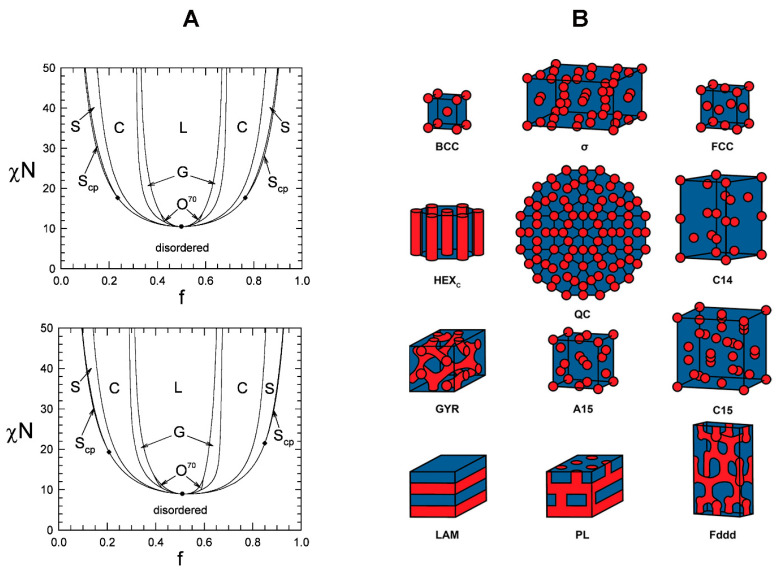
(**A**) The plots display representative phase diagrams of BCP systems determined by the SCFT. The top and bottom plots belong to the phase diagrams of AB diblock and ABA triblock copolymers, respectively. χ is the Flory–Huggins interaction parameter, N is the degree of polymerization, and f is the volume fraction. The symbols of S, S_cp_, C, G, L, and O^70^ denote for body-centered cubic spheres, hexagonally close-packed spheres, cylinders, gyroids, lamellae, and F_ddd_, respectively. Reproduced with permission from Ref. [124] Copyright (2012) American Chemical Society. (**B**) Nanostructures that were determined for linear AB diblock copolymers are schematically depicted. These structures include BCC (body-centered cubic), σ (Frank–Kasper sigma phase), FCC (face-centered cubic), HEX_c_ (hexagonally packed cylinders), QC (dodecagonal quasi-crystal), C_14_ (Frank–Kasper AB_2_ Laves phase), GYR (double gyroid), A_15_ (Frank–Kasper AB_3_ phase), C_15_ (Frank–Kasper AB_2_ Laves phase), LAM (lamellae), PL (perforated lamellae), and F_ddd_ (O^70^ network). Reproduced with permission from Ref. [125] Copyright (2020) American Chemical Society.

The phase separation behaviors of different BCP systems have been extensively studied both theoretically and experimentally [40,45,46,47,49,55,56,57,58,59,60,61,62,63,64,65]. BCP phase diagrams obtained by a self-consistent mean-field and other related theories provide the exact relationship between χN and f which, in turn, dictates the spatial configuration and packing nature of the polymer nanostructures for a given BCP system. For a simple linear A-B diblock whose χN value is greater than ~10.5, ordered nanostructures that range from spheres (body-centered cubic A spheres in a B matrix), to cylinders (hexagonally packed A cylinders in a B matrix), to bicontinuous gyroids (two interpenetrating networks of A and B), and to lamellae (alternating planes of A and B) can be formed depending on the volume fraction and the immiscibility of the polymer blocks. Even a larger collection of nanostructures is available in BCPs composed of triblocks or polymer blocks with higher architectural complexities [56,60,118,119,120,121,122,123]. Figure 1A displays representative phase diagrams obtained by a self-consistent field theory (SCFT) for (a) AB-type diblock and (b) symmetric ABA triblock copolymer systems. For these two-component systems, the BCP morphologies predicted for the ordered state include body-centered cubic spheres (S), hexagonally close-packed spheres (S_cp_), cylinders (C), gyroids (G), lamellae (L), and F_ddd_ (O^70^) [124]. Figure 1B further shows exemplar nanostructures associated with various phases of AB diblock copolymers that were identified by a theory and/or experiment in the literature [125].

One of the crucial aspects of phase diagrams is the characteristic phase separation behaviors of BCPs and therefore, the resulting BCP nanostructures are highly predictable and tunable. The size and shape of BCP domains along with the periodicity between the polymer domains can be readily controlled at the nanoscale level by simply changing experimental variables such as the composition, molecular weight, and volume fraction of the BCP blocks. Moreover, nanostructures that are not thermodynamically stable can be experimentally achieved in certain cases [40,126]. These morphologies arise from additional experimental constraints of kinetic or chemical factors that are applied during the synthesis and fabrication of BCPs. Examples of these factors include heterogeneities in the molecular weight and structure as well as interactions of solvent vapors to select polymer blocks. More detailed discussions will follow in the next section.

### 2.2. Block Copolymer Nanostructures in Thin Films

Surface energetics and confinement effects become extremely important for predicting the phase separation behaviors of BCPs in thin films [41,44,47,62,127,128,129,130]. Therefore, when BCPs are prepared on a solid support, wetting energies associated with polymer–air and polymer–solid interactions are often considered in addition to the thermodynamic parameters previously discussed for bulk phase diagrams. The enhanced role of surface and interfacial energetics as well as the interplay between the BCP film thickness and the equilibrium period of microphase separation can drive a richer array of nanomorphologies than what can be obtained from their bulk counterparts. For example, the formation of BCP nanodomains in thin films can take place in different orientations with respect to the substrate surface. Balancing the energetics between the polymer blocks and the material interfaces above and below the BCP is used to create nanopatterns that are not expected in bulk, specifically in the direction perpendicular to the underlying substrate or in combinations of perpendicular and parallel orientations along the thickness axis. The enthalpic contributions from selective interactions at the top and bottom interfaces are minimized under which condition BCP nanodomains are aligned perpendicular to the substrate due to the entropic contributions of better chain stretching in this direction. Similarly, variations in the polymer–air interfacial energies for the different blocks of a BCP can be used to create chemically alternating nanopatterns at the polymer–air boundary. Nanostructures normal to the underlying substrate can exist partially into the depth of a BCP film. For example, an ultrathin diblock film of polystyrene-block-polymethylmethacrylate (PS-b-PMMA) can be prepared to produce periodic nanopatterns on the film surface where the small difference in interaction energy between PS–air and PMMA–air causes both the PS and PMMA blocks to be exposed at the polymer–air boundary. BCP nanopatterns organized this way can offer distinct chemical properties whose length scale varies at nanoscopic dimensions.

More examples of nanomorphologies can be found in amphiphilic BCP systems, whose structures are first self-assembled in solution and subsequently transferred to a solid substrate [67,126,131,132,133,134,135,136,137,138,139,140]. In these systems, the polymer nanoarchitectures are controlled by the interactions not only between the different polymer segments of a given BCP but also between each polymer segment and the solvent. Amphiphilic BCPs form micellar assemblies in a solution above a critical polymer concentration. For a typical BCP–solvent system, the volume fraction of the polymer segments plays the largest role in determining the morphology of the nanoassemblies. However, their exact sizes and structures can be additionally adjusted by changing the solution properties and environmental parameters such as the pH, temperature, and ionic strength of the solvent as well as the chemical composition, length, and relative solubility of the polymer segments. The most common nanomorphologies found from diblock BCPs are micelles of a spherical and cylindrical (worm-like) shape, although vesicle-shaped micelles (polymersomes) analogous to naturally occurring liposomes are also formed. In triblock BCP as well as nonlinear BCP systems involving hyperbranched polymers, much more complex morphologies such as toroids, helices, and multicompartment micelles have been obtained [131,132,137].

**Figure 2:**
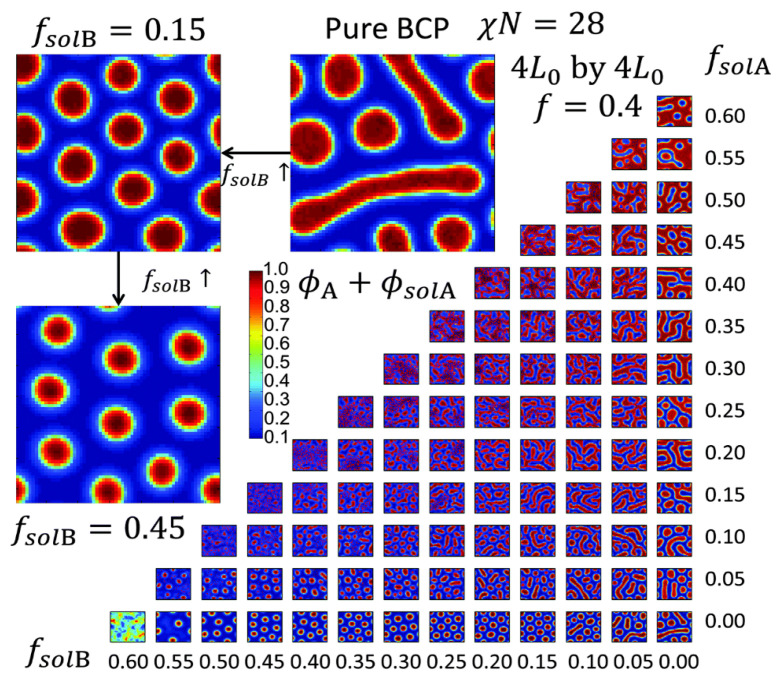
Explicit simulations for solvent vapor annealing were performed in 2D with an AB-type of BCP with f_A_ = 0.4 and a fixed χN = 28 that were exposed to different amounts of block A- and block B-selective solvents with the respective volume fraction of f_solA_ and f_solB_. Various BCP phase regions observed in simulations are then illustrated for various values of f_solA_ and f_solB_. ϕ denotes the local density. L_0_ is the spacing of a set of metastable hexagonally packed cylinders. The 2D bulk morphology with no solvent included nanostructures of circles corresponding to through-plane cylinders and lines corresponding to lamellae of block A. As more f_solA_ was added to the system, the line structures became more dominant and eventually transitioned to a perforated A network surrounding B. As f_solB_ increased, the morphology changed to hexagonally close-packed circles that eventually solvated A-rich micelles. Ordered structures were lost with the increase in both f_solA_ and f_solB_. Reproduced with permission from Ref. [147] Copyright (2015) Royal Society of Chemistry.

The formation of micelles in BCP systems of polystyrene-block-polyacrylic acid (PS-b-PAA), polystyrene-block-polyethylene oxide (PS-b-PEO), polystyrene-block-poly(2-vinylpyridine) (PS-b-P2VP), and polystyrene-block-poly(4-vinylpyridine) (PS-b-P4VP) has been extensively examined [131,137,141,142,143,144,145]. In these systems, it is well-known that additional BCP nanostructures beyond those predicted by thermodynamic considerations can be kinetically isolated via solvent vapor annealing (SVA). This method employs a solvent vapor selective to a particular polymer block which then induces preferential interactions of the selective block to the solvent vapor. A swollen and mobile BCP thin film, upon exposure to the solvent vapor, can result in well-ordered nanostructures on the BCP surface even at a temperature that is well below the glass transition temperature of the polymer blocks. Hence, this method has been widely used to kinetically trap various non-equilibrium, but air-stable micellar nanomorphologies as an avenue to producing periodic nanopatterns on a solid surface. For example, hexagonal micelles of PS-b-P4VP were formed in toluene and prepared into a thin film on a Si support. The PS-b-P4VP thin film was subsequently annealed under the vapor of chloroform. In addition to the original micellar spheres, additional nanostructures that include holes, reformed spheres, embedded spheres, enlarged spheres, cylinder precursors, and cylinders were produced during the solvent annealing process [126,146]. BCP nanostructures that can be generated by controlling solvent vapor annealing have also been identified by computer simulations [147]. For producing different BCP nanostructures, solvent vapor annealing provides additional degrees of freedom such as the fraction of different solvents incorporated, their selectivity with respect to each block, and solvent effects on the surface and interface energies. The effects of these factors on the final nanodomain morphologies of BCPs were examined in 2D simulation studies, as depicted in Figure 2. The simulation results were then compared with the experimental outcomes of polystyrene-block-polydimethylsiloxane (PS-b-PDMS) annealed under block-selective solvents of toluene and heptane [147].

## 3. Block Copolymer Surfaces Interfacing Biomolecules

As discussed so far, BCP self-assembly enables a straightforward and convenient means to obtain patterned nanostructures with high precision and controllability without the need for sophisticated nanofabrication techniques such as extreme UV and electron-beam lithography. Further, self-assembled BCP nanostructures can serve as a powerful and viable platform to position different nanomaterials of interest onto a solid surface with excellent scalability and exquisite nanoscale spatial precision. As such, BCP nanodomains have been well-recognized and utilized as nanotemplates for seeding inorganic nanoparticles (NPs) and as lithographic masks for large-area chemical patterning [42,69,70,144,145,148,149,150].

Another area in which self-assembly plays a crucial role is found in biological systems. Many biological processes and functions rely on the precise positioning and assembly of biomolecules. The spatial positioning and high-level organization of proteins and nucleic acids, for example, inside virus capsids, collagen matrices, and cell membranes occur with nanoscale precision via self-assembly-driven processes. The exact nanoscale arrangements of intricate molecular structures are vital for their proper functions in those cases. It is also imperative to control the assembly of biomolecules in biomaterials for their use in engineered bioplatforms such as medical implant devices, artificial tissue scaffolds, and antibacterial coatings. Likewise, the self-assembly dynamics pertinent to nanoscale organizations of biomolecules onto various surfaces, including those on heterogeneous templates, are critical to controlling protein crystallization, protein printing onto a surface, and timed protein release from a surface. These aspects have direct and important consequences for biosensing and biocharacterization applications.

In comparison to BCP applications in inorganic NP assembly and nanolithography masks, their use in nanoscale bioassembly has not been realized until later [35,36,37,72,75,151,152,153,154,155]. Comparatively speaking, limited work has been undertaken to exploit BCP self-assembly in nanoscale spatial partitioning of biomolecules and their extended assembly on a solid surface. Yet, many intriguing and encouraging discoveries have been put forward so far in this field. The first endeavor in this regard was made by Kumar et al. [75], whose work demonstrated the possibility of creating well-organized protein nanoarrays. Owing to this and ensuing research efforts, it is now well-understood that the spatial assembly of proteins can be faithfully guided not only by the size and periodicity of the nanostructures formed on an underlying BCP surface upon microphase separation, but also by preferential interactions between the different BCP nanodomains and a given protein. This section highlights those research endeavors that successfully utilized self-assembled nanostructures of BCPs as surface guides to derive simple and hierarchical ordering of biomolecules during which processes biomolecules themselves were also organized on the BCP nanostructures via self-assembly.

### 3.1. Proteins

#### 3.1.1. BCP Nanodomains for Proteins: Single-Component Systems

**Figure 3:**
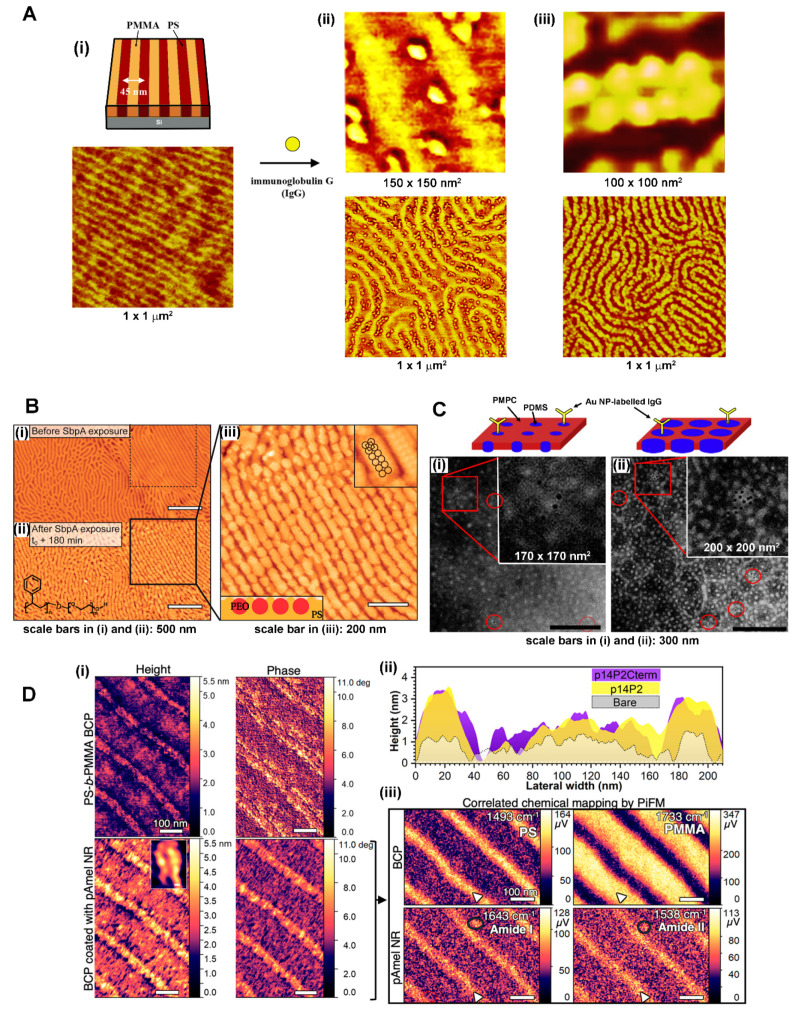
(**A**) The schematic diagram and the AFM panel in (**i**) correspond to the alternating PS (orange) and PMMA (yellow) nanodomains formed on a PS-b-PMMA thin film surface. The repeat spacing of the nanostripes (PS to PS nanodomains) is 45 nm. The AFM images in (**ii**,**iii**) display the exclusive interaction of IgG molecules (appearing as spheres) with the more hydrophobic PS block of PS-b-PMMA under different protein loading conditions. In all cases, the distribution of IgG molecules was consistently observed to be segregated only on the PS nanodomain areas of the BCP surface. The AFM data in (**iii**) belong to an IgG monolayer-forming condition under which all available PS nanodomains were fully occupied by densely packed IgG molecules. Two IgG molecules assembled along the short axis of the PS nanodomains at maximum due to the protein size with respect to the width of the underlying PS nanodomain. The assembly of IgG molecules on the BCP surface resembled the packing nature found in a 2D protein crystal. Adapted with permission from Ref. [75] Copyright (2005) American Chemical Society. (**B**) A blank template of nanostriped PS-b-PEO thin film is displayed in (**i**) onto which SbpA was incubated. The treatment resulted in the formation of S-layer crystals confined to the PS regions of the PS-b-PEO surface, as shown in the AFM panel of (**ii**). The internal crystal structure of the S-layer is shown in the magnified image of (**iii**). Adapted with permission from Ref. [82] Copyright (2019) American Chemical Society. (**C**) The TEM data display the surface of PMPC-b-PDMS after treating the surface with a solution of AuNP-labelled IgG. The PMPC-b-PDMS surfaces used in (**i**,**ii**) contained a PDMS monomer unit composition of 40.7% and 55.4%, respectively. Cylindrical PDMS domains were produced in a PMPC matrix with different domain sizes as schematically shown in (**i**,**ii**). Small dark dots inside the red circles in the TEM panels correspond to the AuNP-labelled IgG molecules segregated on the more hydrophobic PDMS regions of the BCP surface. Adapted with permission from Ref. [81] Copyright (2009) Elsevier. (**D**) The AFM results in (**i**,**ii**) and PiFM data in (**iii**) show the peptide analogues of pAmel NRs assembled on 50 nm PS stripes of PS-b-PMMA. The top and bottom panels in (**i**) correspond to the blank template of PS-b-PMMA and after incubation with p14P2, where the inset in the bottom left panel (scale bar of 10 nm) displays the morphology of two pAmel NRs on PS. Height profiles measured perpendicular to the stripes are compared among the cases of the bare BCP, p14P2-coated BCP, and p14P2Cterm-coated BCP in (**ii**). The PiFM surface maps of p14P2-coated BCP in (**iii**) were obtained at the excitation wavelength specific to the BCP as well as to the β-sheet pAmel NRs. The specific wavelength used is marked in each map and the arrows point to the region of excitation. The strong signals appearing as bright stripes in the PiFM data are from pAmel NRs with a β-sheet conformation which were assembled on the PS regions. The relatively low signal of the PMMA areas in the Amide I and II maps indicated the lack of β-sheet NRs. Adapted with permission from Ref. [83] Copyright (2023) American Chemical Society.

**Protein Interactions on BCP Thin Films.** The spearheading study of protein nanoarrays guided by an underlying BCP surface of PS-b-PMMA demonstrated that individual protein molecules self-assemble on BCP nanodomains via preferential protein–PS interactions [75]. A model protein of immunoglobulin G (IgG) was successfully ordered on the PS nanodomain areas of PS-b-PMMA. Figure 3A displays such exclusive interaction behavior of IgG with the PS nanodomains that was unambiguously resolved at the individual protein level on the BCP nanodomain surface. It is clear from the atomic force microscopy (AFM) data that the surface partitioning of IgG molecules was entirely exclusive to the PS nanodomains, leaving the PMMA nanodomains completely free of IgG. This was due to the preferential interaction of IgG with the more hydrophobic block of PS relative to PMMA. The different degree of IgG loading on the BCP surface in Figure 3A was controlled by adjusting the bulk solution concentration of IgG and incubation time on the surface. When the loading condition was tuned to a monolayer-forming coverage, all available PS sites were packed densely with a single layer of adsorbed IgG. The packed protein layer on the PS nanodomains contained two IgG molecules along the short axis of the nanodomain direction, as shown in the rightmost panels of Figure 3A. This was because the width of the underlying PS domains used for the study was commensurate with approximately two IgG molecules assembled side by side along the short nanodomain axis. The study was the first demonstration of a BCP thin film-based approach for achieving nanopatterned proteins on a solid surface, while solely relying on the self-assembly processes of the BCP as well as the biomolecules.

In many other stimulating studies following this work, other proteins, largely globular in shape, were able to be similarly assembled into nanopatterns [35,37,79,81,82,86,95,151,156,157]. Proteins and peptides such as human and bovine serum albumins (HSA and BSA), horseradish peroxidase (HRP), mushroom tyrosinase (MT), green fluorescent protein (GFP), protein G (PG), and amelogenin (Amel) behaved similarly as IgG on a PS-b-PMMA thin film. Much like the data presented in Figure 3A, the assembled protein patterns under a monolayer forming condition faithfully followed the size and shape of the more hydrophobic BCP nanodomains [35,37,79,81,82,83,86,95,151,156,157]. The AFM, transmission electron microscopy (TEM), and photo-induced force microscopy (PiFM) data in Figure 3B through 3D display some of these examples. As shown in Figure 3B, S-layer protein (SbpA) treated on a nanostriped BCP thin film of polystyrene-block-polyethylene oxide (PS-b-PEO) yielded the formation of S-layer crystals confined to the more hydrophobic PS block of the BCP surface. The TEM data in Figure 3C correspond to gold nanoparticle (AuNP)-labelled IgG that segregated into the more hydrophobic PDMS block on the thin film surface of poly(2-methacryloyloxyethyl phosphorylcholine)-block-poly(dimethylsiloxane) (PMPC-b-PDMS). The AFM and PiFM results in Figure 3D present the assembly of the peptide analogues of phosphorylated amelogenin (pAmel) nanorods (NRs) on the PS stripes of PS-b-PMMA when the BCP surface was incubated with p14P2. The pAmel NRs on the PS-b-PMMA thin film were formed via the self-assembly of p14P2 (GHPGYINF p(S) YEVLT) and p14P2Cterm (GHPGYINF p(S) YEVT DKTKREEVD) on the more hydrophobic PS block.

**Elongated Protein Interactions on BCP Thin Films.** It was also demonstrated that the size and periodicity of the BCP nanodomains could be further utilized to modulate the surface partitioning of elongated proteins into nanoscopic patterns upon their self-assembly on the BCP surface [84]. It was revealed that, for a system of an elongated protein of fibrinogen (Fg) on PS-b-PMMA, the interaction differences between the two polymer blocks as well as those between the D, E, and αC subunits within a Fg molecule can lead to protein concentration-dependent and protein subunit-specific behaviors of Fg partitioning on the BCP surface. Individual Fg molecules show high aspect ratios of ~10 (length to width) and ~25 (length to height). Unlike the globular protein case discussed above, the interaction of Fg to the PS nanodomains was less exclusive where, depending on the protein concentration, Fg showed a more neutral tendency for shared interactions with both blocks of PS and PMMA. The interaction forces governing Fg were found to arise from not only hydrophobic but also electrostatic in nature. This is different from the globular protein interactions discussed earlier which were dominated by the hydrophobic interactions.

**Figure 4:**
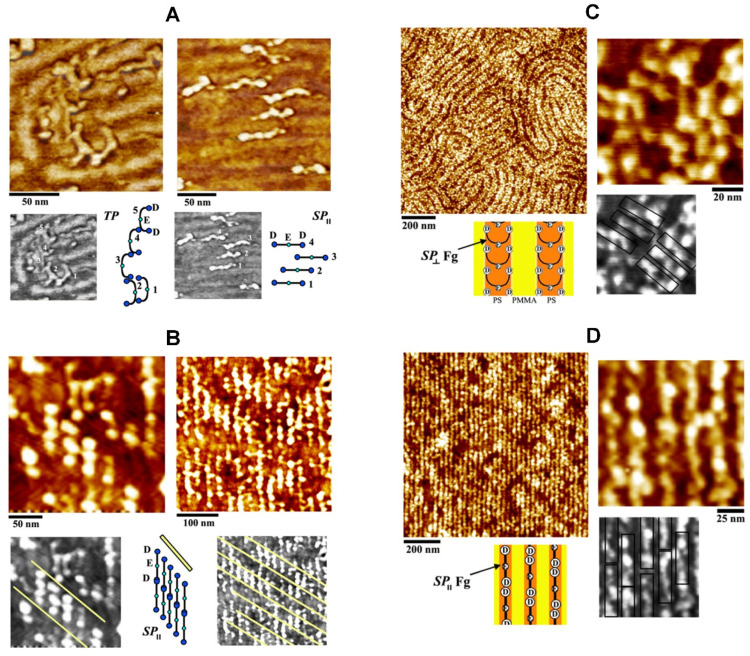
The AFM panels display different Fg configurations on various PS-b-PMMA surfaces. TP and SP refer to the Fg configuration for which the backbone of a Fg molecule lies both on the PS and PMMA nanodomain areas (TP) and only on the PS regions (SP). *SP*_∥_ and *SP*_⊥_ denote the backbone of a Fg molecule lying along the long (*SP*_∥_) and short (*SP*_⊥_) axis of the PS nanodomains. The exact Fg configuration and large-area assembly on PS-b-PMMA were dependent not only on the protein concentration but also on the periodicity and alignment degree of the underlying BCP nanotemplate. (**A**) Mixed populations of Fg molecules with TP and *SP*_∥_ were found on unaligned PS-b-PMMA nanodomains of 25 nm in repeat spacing. The cartoons inserted next to each AFM panel depict the inter- and intra-molecule arrangements of the different Fg subunits of D and E. (**B**) On a PS-b-PMMA surface with fully aligned nanodomain of 28 nm in periodicity, all Fg molecules assembled on the PS nanodomain areas in the direction parallel to the long axis of the PS nanodomains (*SP*_∥_). The yellow lines show the characteristic slope formed by the neighboring Fg molecules. (**C**) Fg molecules assembled on the PS areas in the orientation parallel to the short axis of the PS nanodomains (*SP*_⊥_ configuration, side-on packing) when a PS-b-PMMA surface with 45 nm in periodicity was used to form a monolayer of Fg molecules. Black boxes mark individual Fg molecules. (**D**) On a PS-b-PMMA surface with fully aligned nanodomains of 28 nm in periodicity, Fg molecules under a monolayer forming condition occupied the PS areas in the orientation parallel to the long axis of the PS nanodomains (*SP*_∥_ configuration, end-on packing). Reproduced with permission from Ref. [85] Copyright (2016) American Chemical Society.

Figure 4A presents such complex interaction behaviors observed from the elongated protein of Fg on a PS-b-PMMA surface. Mixed populations of Fg molecules with TP and SP configurations were observed on the BCP surface that contained unaligned nanodomains with a repeat spacing of 25 nm. TP and SP stand for the configuration of Fg on the BCP surface, where the entire length of a Fg molecule (~48 nm in length) lies across the PS and PMMA nanodomain areas (two phases, TP) versus only within the PS nanodomain areas (single phase, SP). The study also revealed surface-specific Fg conformations on the BCP as well as on the homopolymer surface consisting of PS or PMMA. In addition, BCP surface-driven topological changes of single proteins were experimentally resolved for the first time at the sub-biomolecule level. Figure 4B presents such BCP surface-driven effect on the assembly of Fg molecules. Compared to the data on an unaligned nanodomain template in Figure 4A, Figure 4B displays Fg molecules on fully aligned nanodomains of a PS-b-PMMA substrate. The aligned BCP template used for Figure 4B had a repeat spacing comparable to the unaligned sample of Figure 4A. All populations of Fg molecules on the aligned BCP exhibited the SP configuration.

In a later study, the effects of BCP periodicities and alignments on Fg interactions were scrutinized [85]. Different PS-b-PMMA substrates that contained fully aligned or randomly oriented nanodomain of varying sizes were employed. The length scale of the nanodomains between the samples was varied to exhibit a dimension that was much larger, comparable to, and much smaller than the length of Fg. The adsorption behaviors of several Fg molecules in isolation as well as the assembly of many Fg molecules in large-area surface packing were further investigated on the different BCP substrates. The study reported that the periodicity and orientation of the chemically alternating BCP nanodomains can be exploited to manipulate the packing configuration of Fg molecules on the BCP surface. For example, an end-on (side-on) packing geometry, where the backbone of Fg is parallel (and perpendicular) to the long axis of the PS nanodomain, can be achieved by providing a BCP template with a nanodomain periodicity much smaller than (compatible to) the length of the protein. Figure 4C,D summarize these results. The application of a PS-b-PMMA template with a repeat spacing of 45 nm (comparable to the length of a Fg molecule) versus 28 nm (much smaller than the Fg length) for the protein assembly was able to induce side-on (Figure 4C) versus end-on (Figure 4D) packing of Fg molecules on the PS nanodomain areas. Highly oriented nanostructures formed on melt-drawn, ultrahigh molecular weight polyethylene (UHMWPE) surfaces were also shown to induce Fg assembly [158]. Similar to the nanostructures on the BCP surfaces, the nanocrystalline lamellae on the UHMWPE surface were able to control the conformation and aggregation of human plasma Fg. The lateral orientational order of proteins on the polymer surface was dependent on multiple parameters such as nanoscale topography, chemistry, crystallinity, and molecular chain anisotropy of the UHMWPE surfaces.

These works showed that the structural and chemical features of BCP and related polymer surfaces could be effectively used to control not only the spot size and periodicity of the assembled proteins at the nanometer range, but also the orientation and packing geometry in the large-area organization of elongated proteins. Controlling the spatial arrangement of Fg molecules on solid surfaces has important biomedical relevance to blood clotting and wound healing since a specific arrangement of Fg molecules is required in these processes [159]. As evidenced in Figure 4B,D, the BCP-generated Fg nanoassemblies produced Fg molecules in a half-staggered manner, yielding protofibrils of Fg molecules arranged in different PS nanodomains. The half-staggered packing of Fg molecules enabled contact points for the D-E subunits between neighboring Fg molecules on adjacent PS nanodomains. This intermolecular assembly pattern of Fg molecules is similar to the natural process of fibrin assembly in blood clotting. Such an aspect will be important for future applications of BCP-based protein nanoassemblies in developing biomaterials.

#### 3.1.2. BCP-Guided Protein Assembly on Extended Systems Involving Various BCP Thin Films and Proteins

Other studies have since demonstrated that a BCP-based method can be effectively used to attain a large-scale surface organization of biomolecular nanopatterns in a controllable and predictable manner [78,81,85,86,87,88,90,91,95,96,97,103,126,140]. Diblock and triblock copolymer systems used for protein assembly have been extended to include a range of different BCP blocks such as polystyrene-block-polyisoprene (PS-b-PI), polyethylene glycol-block-polystyrene (PEG-b-PS), poly(2-methacryloyloxyethyl phosphorylcholine-block-poly(dimethylsiloxane) (PMPC-b-PDMS), polystyrene-block-poly(2-hydroxyethyl methacrylate) (PS-b-PHEMA), poly(acrylic acid)-block-poly(N-isopropyl acrylamide) (PAA-b-PNIPAM), PS-b-PEO, PMMA-b-PHEMA-b-PMMA, and PAA-b-PMMA-b-PAA. In addition, a diverse system of whole proteins, protein fragments, protein coats, peptides, and extracellular matrix (ECM) fragments has been employed as a model biomolecule for BCP-based self-assembly. Regardless of the BCP and protein model systems used, it was possible to effectively modulate selective adsorption, morphology, orientation, and alignment of proteins on the BCPs. This was achieved by tuning the underlying BCP nanostructures to favorably recognize the different physicochemical properties of the proteins.

**Preferential Protein Interaction with the Hydrophobic BCP Domains.** Many studies reported that proteins and protein coats preferentially interact with the more hydrophobic segments of BCPs [81,82,86,95]. An example of this can be found in a study that employed highly oriented lamellar nanopatterns of PS-b-PMMA as a platform to assemble nanopatterns of various serum, antithrombogenic, as well as cell adhesive proteins such as γ-globulin, Fg, fibronectin (FN), thrombomodulin (TM), and type I collagen (Col I) [86]. The preparation of the BCP thin film was formulated to have a perpendicularly oriented, lamellar morphology of alternating PS and PMMA regions on the surface. The lamellar structures were then aligned along the thickness gradient for producing unidirectional protein nanopatterns of γ-globulin molecules, FN, and TM on the hydrophobic PS areas. Unlike these proteins, Col I molecules did not show any particular orientation on the BCP template, but almost all of the adsorbed parts of Col I were reported to interact with the PS domains. In a different study involving a polymer blend surface, the important roles that the size and surface coverage of polymer heterogeneities play in modulating the diameter and length of Col I assemblies have been identified [160]. When a blend thin film consisting of PS and PMMA was employed, the organization of Col I was reported to be affected by the size of the PS areas in the blend film. The study also confirmed that the amount of Col I adsorbed on the surface was linearly correlated with the PS surface fraction of the blend film.

The general tendency of proteins favoring the more hydrophobic domain of BCPs was further confirmed by examining the adsorption behaviors of BSA, FN, and crystalline surface layers (S-layer crystals) on the BCP surfaces of PS-b-PI, PMPC-b-PDMS, and PS-b-PEO [81,82,92,95]. Various nanopatterns and surface chemistry of solvent-annealed PS-b-PEO were found to efficiently steer the formation of crystalline S-layers from monomeric SbpA confined to the PS nanodomains of the BCP surface [82]. A study using well-ordered, nonequilibrium nanostructures of PS-b-PI also reported that BSA as well as FN tended to bind selectively on the PS domains of PS-b-PI [92,95]. The overall patterns produced by the BSA or FN molecules were found to closely resemble the nanopattern shape of the underlying PS-b-PI nanostructures. Phase-separated BCP surfaces composed of PMPC-b-PDMS were shown to exhibit selective binding of FN molecules to the hydrophobic PDMS domains as well [81].

**Figure 5:**
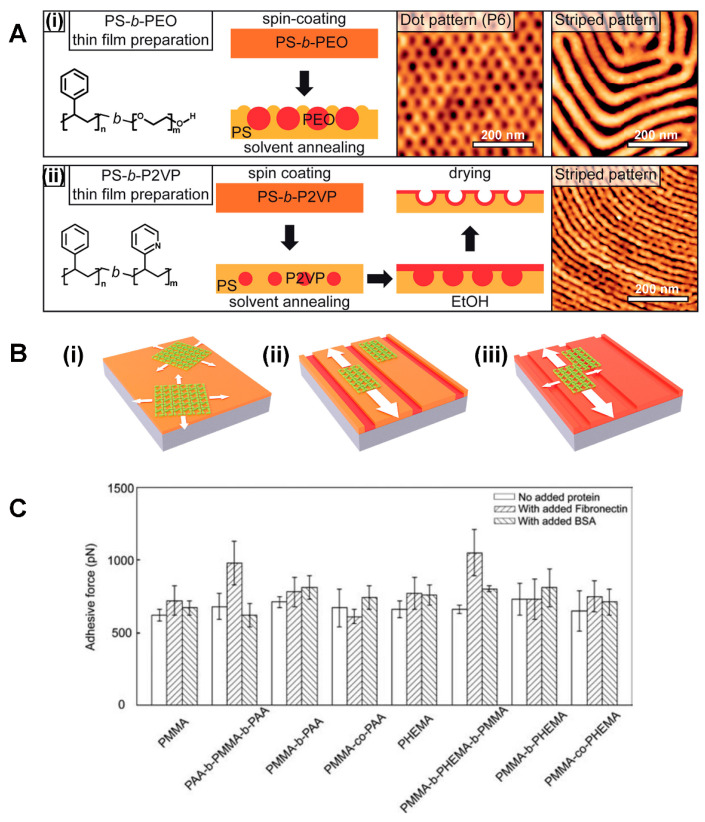
(**A**) The BCP surfaces of (**i**) PS-b-PEO and (**ii**) PS-b-P2VP prepared to examine the effects of structural and chemical contrasts on protein organization are displayed. Relative to the PS-b-PEO surface, the patterned PS-b-P2VP presented a comparable physical contrast but lacked a chemical contrast in terms of alternating hydrophobicity and hydrophilicity. (**B**) The illustrations depict different S-layer formation processes due to the surface effects of structural and chemical contrasts. Isotropic nucleation and growth are expected for S-layers on a uniform surface in (**i**). On a nanopatterned surface with alternating hydrophobic and hydrophilic domains in (**ii**), S-layers nucleate and preferentially grow on the hydrophobic regions only. Lastly, on a nanopatterned surface with no chemical contrast in (**iii**), S-layers nucleate equally on both nanodomains although its growth rate is faster along the long axis of the nanodomain. (**A**,**B**) Adapted with permission from Ref. [82] Copyright (2019) American Chemical Society. (**C**) The bar diagram summarizes measured adhesive forces between antibody-functionalized tips and various polymer surfaces with and without added protein. In both series of BCP templates containing the more hydrophilic (PMMA and PAA) and the less hydrophilic (PMMA and PHEMA) blocks, it was the triblock copolymers that exhibited the highest adhesive force. Adapted with permission from Ref. [90] Copyright (2012) Wiley Periodicals, Inc.

**Topographical versus Chemical Contrast on a BCP Surface for Protein Assembly.** PS-b-P2VP and PS-b-PEO were employed as model BCP systems in a research effort to discern the BCP effects of a structural (i.e., topographic) versus chemical origin on protein assembly [82]. Figure 5A displays the preparation processes of these BCP thin films for the assessment of structural versus chemical effects. The two BCP surfaces were prepared to have a topographic contrast similar to each other, while presenting different chemical contrasts in terms of alternating hydrophobicity and hydrophilicity. The PS-b-P2VP surfaces, uniformly hydrophilic relative to PS-b-PEO in terms of their chemical contrast, resulted in no confinement of the S-layer crystals to any specific nanodomains. Figure 5B schematically illustrates the different formation processes of the S-layer due to the structural and chemical variations associated with the underlying polymer substrates. It was concluded that the presence of a chemical contrast on the PS-b-PEO template played a critical role in the spatially confined assembly of the crystalline S-layers [82].

The effects of BCPs’ structural and chemical contrasts on protein assembly have been further examined not only on diblock but also on triblock and other related polymer systems. The different roles of the BCP’s structural and chemical effects have been studied by measuring the adhesion forces of proteins on surfaces. Diblock and triblock as well as random copolymers consisting of PMMA, PAA, and PHEMA were evaluated for their differences in FN interaction [90]. While keeping PMMA as one of the blocks, the other two polymers were used as varying segments. Different diblock and triblock surfaces such as PMMA-b-PAA, PMMA-b-PHEMA, PMMA-b-PHEMA-b-PMMA, and PAA-b-PMMA-b-PAA were prepared this way. It was found that the surface distribution of FN molecules was dictated by both the chemical effect stemming from the interactions between FN and the polymer chain of PMMA, PAA, or PHEMA, and the topographic effect due to the nanoscale dimension and spacing of the polymer domains. The conformation and orientation of FN were determined by the surface chemistry as well as the nanomorphology of the BCP templates. However, the study pointed out that the adhesion forces between FN on the BCP surfaces and FN antibody hanging from a probe tip did not depend either on the chemistry, charges, or wettability of the BCP surfaces. Rather, the adhesion forces between FN-FN antibodies were governed by the BCP nanomorphology. In general, higher adhesion was monitored for the triblock surfaces that presented a larger domain size relative to the diblock samples. This tendency was consistently observed whether the sample surfaces contained the more hydrophilic (PMMA and PAA blocks) or the less hydrophilic (PMMA and PHEMA blocks) polymer segments. Figure 5C summarizes the measured adhesion forces between the different polymer surfaces and antibody-functionalized tips.

**Stimuli-Responsive BCP Segments for Protein Interactions.** Protein behaviors at surfaces have been successfully tuned with the aid of stimuli-responsive segments in BCPs. A BCP thin film of PAA-b-PNIPAM, assembled in a layer-by-layer manner, was employed to study the adsorption behaviors of ovalbumin (OVA) while varying the temperature and the pH of the protein solution [96]. The pH-responsiveness of the PAA block and the thermo-responsiveness of the PNIPAM block provided a dual sensitivity to modulate protein interactions at the PAA-b-PNIPAM surface. It was found that OVA adsorption to the BCP surface was dependent on the temperature. The BCP film exhibited high OVA adsorption at 50 °C whereas the same BCP surface strongly repelled the protein at 20 °C.

**Micellar BCP Inversion in Protein Assembly.** Tuning protein behaviors at the surface has been attempted by flipping the spatial arrangement of the polymer segments belonging to the core and matrix (corona) portions of BCP nanostructures as well. Heterogeneous nanopatterns assembled from an amphiphilic BCP of PS-b-PHEMA were used for Fg adsorption [88]. The study found that the protein-adhesive/-resistant property of the underlying surface can be tuned by switching out the core and matrix polymer components of the heterogeneous nanopatterns. When the PS-b-PHEMA surface was processed to yield PHEMA (PS) domains to occupy the majority (minority) of the surface, the film became strongly protein-repulsive. In contrast, the opposite distribution of majority PS and minority PHEMA domains on the film surface led to protein adsorption.

**Chemical Modifications of BCPs and Proteins for Specific Interactions.** Strategies to chemically modify the nanodomains of a specific BCP block as well as proteins of interest have been used to induce exclusive polymer block–protein interactions on a BCP surface. In one study, a biotinylated BCP surface of PEG-b-PS was prepared into cylindrical nanostructures by mixing a small amount (4 mol%) of biotin-functionalized BCP into non-functionalized BCP [97]. Upon subsequent incubation with streptavidin (SAv) on the biotinylated BCP surface, the strong interaction between biotin and SAv led to the immobilization of SAv to the PEG-b-PS thin film. The protein immobilization was controlled by varying the amount of biotinylated PEG-b-PS used for mixing. In another study, alkyne-functionalized BCP nanopatterns of PS-b-PHEMA were demonstrated for linking azide-tagged protein molecules of Fg, myoglobin (Mb), and lysozyme (LZM) [89]. The azide-tagged protein molecules bound to the alkyne-functionalized PS nanodomains of PS-b-PHEMA. The approach was able to conveniently produce nanoarrays containing individual protein molecules per spot via specific protein binding to the PS nanodomains and eliminated any issues in protein quantification that might arise from nonspecific protein adsorption. In addition, a chemically modified BCP of PS-b-PEO was self-assembled to produce the functional group of maleimides on the PEO nanodomains while controlling the size, number density, and lateral spacing of the nanodomains [91]. The maleimide group was then employed to bind proteins and extracellular matrix (ECM) fragments such as GFP, FN fragments, and arginine-glycine-aspartate (RGD)-containing peptides on the PEO domains. The study also showed that the same maleimide-functionalized BCP templates were applicable for linking other biomolecules such as poly-histidine tagged proteins and Zn-chelating peptide sequences.

**Protein Embedded in BCP Thin Films.** Attempts to create hierarchically structured, functional biomaterials have been made by directing co-assembly of BCP thin films and biomolecules of proteins or peptides. These platforms were also used to carry out a quantitative examination of the release kinetics of biomolecular cargos within BCP thin films. PS-b-PEO thin films prepared into different thicknesses were co-assembled with cargo proteins and peptides such as LZM and a peptide of TAT [99]. This process led to the distribution of protein or peptide cargos within the hexagonally packed PEO nanodomains of PS-b-PEO. In a different study, the co-assembly method was extended to build assembled structures of a greater hierarchy [98]. Structures consisting of PS-b-PEO and a bio-motif were designed for a simultaneous co-assembly scheme. Bio-motifs such as horse-heart Mb and a heme-binding protein were used in the co-assembly to produce protein/cofactor complexes as well as catalytically active enzymes within the BCP thin film. In another endeavor, a solvent-induced film of hexagonally packed PS-b-PEO nanostructures was processed into vertically arranged, cylindrical nanoscaffolds for the assembly of Lsmα [102]. Lsmα is a protein that self-organizes into stackable, doughnut-shaped, heptameric structures whose pore size can be tuned for encapsulation molecules of interest. Upon co-assembly of PEGylated Lsmα (LsmαPEG) with PS-b-PEO in a solvent mixture composed of water, methanol, and benzene, the protein molecules were able to form into a regular array. The assembled array structure contained doughnut-shaped tunnels of Lsmα. The work showed that BCP templates can effectively guide even a coordinated assembly of hierarchical protein nanostructures into BCP nanodomains, beyond what has been demonstrated for the assembly of simple proteins on BCP nanodomains. All these efforts will be crucial for the future applications of biocargo-loaded BCP films in cell culture and mechanotransduction studies as well as in biocatalytic reactions and biosensing.

**Nanoporous BCP Thin Films for Protein Assembly.** Nanoscale protein interactions with BCPs have been extended to those with nanoporous thin film structures [104,161,162,163]. Nanopores in self-assembled BCP thin films are typically produced by selectively removing a polymer block from a phase-separated BCP film [163]. Methods used for the selective segment removal include ultraviolet (UV) degradation, reactive ion etching (RIE), ozonolysis, and chemical etching [164,165,166,167]. The resulting size and shape of the nanopores in the BCP templates are governed by the original nanostructures formed during the BCP’s phase separation process and, thereby, the nanoporous structures can be controlled by the same experimental parameters that are used to modulate the BCP nanodomains according to their phase diagrams. In a study using the nanopore approach, a thin film of nanometric channels was fabricated from a BCP mixture of polystyrene-block-poly(l-lactide) (PS-b-PLLA) and PS-b-PEO [104]. Nanoporous structures with elongated nanopores of ~20 nm in width were generated after the selective removal of PLLA from the phase-separated BCP mixture. The resulting nanochannels contained PEO chains pending from PS walls. The BCP nanopore thin film was then successfully utilized for the immobilization of HRP molecules.

**Figure 6:**
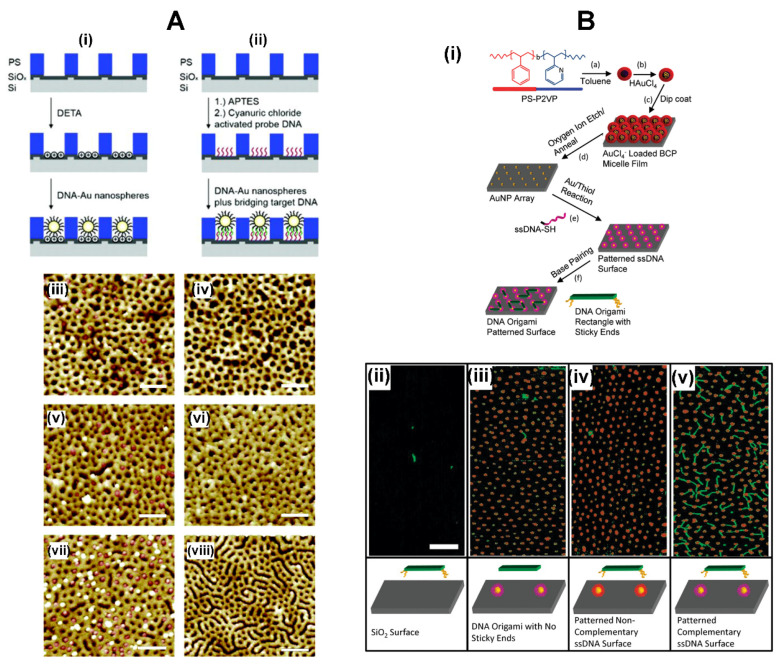
(**A**) The schematic representations show the processes for Au-DNA functionalization via (**i**) DETA and (**ii**) APTES on nanoporous PS-b-PMMA thin films. DETA and APTES denote (3-trimethoxysilylpropyl)-diethylenetriamine and (3-aminopropyl)-trimethoxysilane, respectively. The AFM images correspond to the DETA-functionalized (**iii**,**v**,**vii**) and unfunctionalized (**iv**,**vi**,**viii**) nanoporous BCP templates exposed to DNA-AuNPs. Red circles inserted in the images are the DNA-AuNPs that were deposited into the DETA-functionalized nanopores. All scale bars are 200 nm in size. Adapted with permission from Ref. [107] Copyright (2006) American Chemical Society. (**B**) The schematic in (**i**) displays the patterning process of AuNPs on PS-b-P2VP micelles for the adsorption of DNA origami on the BCP surface. The AFM panels show (**ii**) sticky end-modified, DNA origami placed on a clean SiO_2_ surface, (**iii**) non-modified DNA origami on a patterned, single-strand DNA (ssDNA) surface, (**iv**) sticky end-modified DNA origami on a patterned, noncomplementary ssDNA surface, and (**v**) modified DNA origami on a patterned, complementary ssDNA surface. All scale bars are 200 nm in size. Adapted with permission from Ref. [108] Copyright (2011) American Chemical Society.

**Indirect BCP–Protein Interactions via Inorganic Nanoparticles.** Research efforts have been made to assemble biomolecules at BCP surfaces through a mediating layer of inorganic NPs using a process known as BCP micelle nanolithography, instead of having BCP nanopatterns directly interface with proteins on the polymer surfaces [68,105,106,107,108]. In these works, inorganic NPs such as gold NPs (AuNPs) of 1–15 nm in size were pre-assembled on BCP nanoguides with a tunable lateral spacing of 15–250 nm through a preferential metal–polymer segment interaction. Well-defined patterns of AuNPs were subsequently produced after subjecting BCP thin films to a plasma process to remove the BCP from the substrate, leaving only the AuNPs. As the lateral spacing in the BCP template can be controlled by the BCP molecular weight, the periodic spacing between AuNP dots in the array can be adjusted accordingly. Fabrication processes similar to those depicted in the schematics of Figure 6A are typically used to create inorganic NP-linked templates via the BCP micelle nanolithography. The NP-containing BCP surfaces can then be used for assembling DNA, peptides, or proteins.

In studies using the micelle nanolithography method, AuNPs with tunable sizes were generated into a quasi-hexagonal pattern by employing a sacrificial PS-b-P2VP template [105,106]. The AuNP array was further used to assemble thiolated αvβ3 integrin receptor of c(-RGDfK-) functionalized through thiol–Au interactions. Nanoporous structures fabricated from PS-b-PMMA were also utilized for the deposition of DNA-conjugated AuNPs [107]. As illustrated in Figure 6A, nanopatterns on the PS-b-PMMA thin film were first exposed to UV radiation to cross-link the PS chains while degrading PMMA. Nanopores were then created on the PS-b-PMMA surface by rinsing away the degraded PMMA with a solvent. The resulting nanopores on the BCP surface were able to serve as nanocontainers for the AuNPs whose NP surfaces were pre-conjugated with oligonucleotides. Similarly, PS-b-PMMA and PS-b-P2VP surfaces were fabricated to produce AuNP and Au nanorod (AuNR) arrays of various diameters and center-to-center distances [68,108]. The Au-modified BCP templates were used afterwards to selectively place DNA origami at directed surface locations. An example of such efforts involves AuNPs and AuNRs formed on the hexagonal array of PS-b-P2VP micelles [108]. The Au-containing BCP surface was functionalized with thiol-modified, single-stranded DNA (ssDNA-SH). DNA origami created with sticky ends was then attached to the surface by extending appropriate staple strands on each end. The modified staple strands connected to DNA origami subsequently pair up with the ssDNA-SH. The AFM results from the AuNP-modified PS-b-P2VP micelles compared to those of control surfaces are shown in Figure 6B.

**Protein Adsorption and Release Kinetics on BCP Thin Films.** It has been revealed that the time-dependent adsorption behaviors of proteins differ on nanoscale BCP surfaces when compared with those on the surfaces of homopolymer counterparts. So far, investigations of proteins on nanoscale polymer surfaces have been largely centered on static instead of time-dependent behaviors. This is mainly due to the experimental challenges associated with directly attaining single biomolecule imaging and kinetic data. Being able to experimentally identify key kinetic segments that can be substantiated by corresponding topological data will be critical. However, the measurement process becomes especially difficult for the very early stage of protein adsorption, where ensemble-averaged measurement techniques may not be adequate for correctly rendering the kinetics associated with single biomolecule behaviors at nanoscale surfaces.

**Figure 7:**
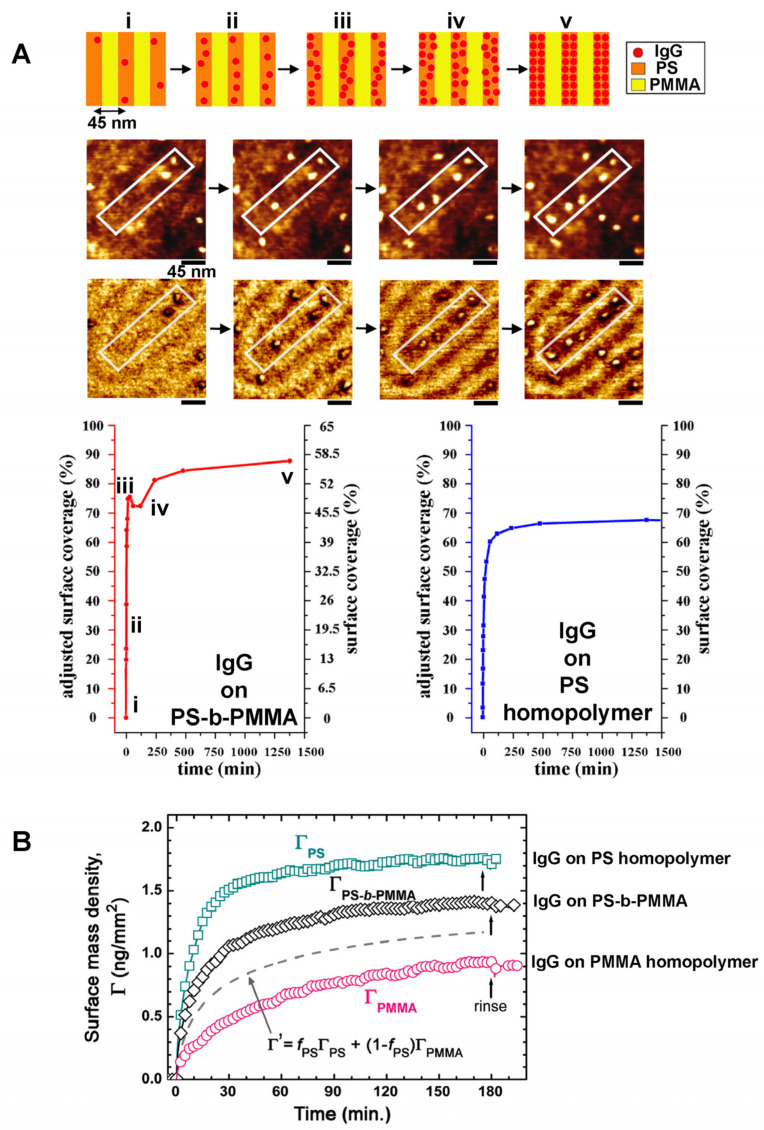
(**A**) The AFM data and IgG adsorption isotherms were obtained by time-lapse imaging of the same PS-b-PMMA surface areas while tracking individual IgG molecules. As a guide, white boxes are inserted in the time-lapse AFM data to mark the same BCP area. Key kinetic segments identified from the IgG assembly on the BCP surface are linked to the topographic data corresponding to each segment specified as (**i**–**v**). Unlike the IgG behavior on the control surface of PS homopolymer, IgG adsorption isotherms presented two unique features at the nanoscale BCP surface, i.e., the presence of two Langmuir-like segments and the existence of an undulating, nonmonotonic adsorption regime. Adapted with permission from Ref. [76] Copyright (2022) American Chemical Society. (**B**) The plot of surface mass density versus time corresponds to IgG adsorption on the BCP surface of PS-b-PMMA as well as on the homopolymer control surfaces of PS and PMMA. The data were obtained by SPR spectroscopy. The dashed line shows the hypothetical amount of adsorbed IgG, which was calculated from the weighted average of PS and PMMA homopolymer data while considering the volume fraction of the two polymer blocks in the BCP. Adapted with permission from Ref. [77] Copyright (2009) American Chemical Society.

Despite these hurdles, the exact adsorption pathways and kinetics of IgG were determined successfully by tracking individual IgG molecules on the striped nanodomains of PS-b-PMMA [76]. Owing to the direct measurements of the same IgG molecules over time, it was possible to establish meaningful correlations between various topological states of the IgG assembly and specific adsorption kinetic regimes on the BCP surface. These characteristics were then compared to those data similarly acquired on a PS homopolymer surface. A distinct adsorption pathway of a single to double-file IgG assembly was revealed on the BCP surface. Additionally, unique adsorption characteristics such as the presence of two Langmuir-like segments and an undulating nonmonotonic regime were identified on the nanoscale BCP surface [76]. On the control surfaces of PS and PMMA homopolymers, the kinetic profile of IgG adsorption exhibited a single Langmuir-like segment with no undulating regime [76,77]. The IgG adsorption kinetics on the BCP surface of PS-b-PMMA versus on the homopolymer surfaces of PS and PMMA are presented in Figure 7A,B. Data in Figure 7A were collected from single protein tracking by AFM in a time-lapse manner, and those in Figure 7B were obtained by surface plasmon resonance (SPR) spectroscopy.

In addition to adsorption kinetics, the release kinetics of single-component proteins from BCP surfaces were also examined [99]. The release kinetics of fluorescein isothiocyanate isomer (FITC)-coupled protein of LZM as well as FITC-coupled peptide of TAT from PS-b-PEO thin films were measured by spectrofluorometry [99]. By taking advantage of the fact that BCP film thickness can be easily modulated during the spin coating process of the film preparation, PS-b-PEO samples of 45–60 nm in thickness were prepared. The thinnest (thickest) BCP film yielded the least (greatest) amount of released protein cargo. Quantitatively, 20–80 ng cm^−2^ of cargo was reported to be released from PS-b-PEO films, where the larger (smaller) molecule of LZM (TAT peptide) was released over a longer (shorter) period. As for the release kinetics of the biomolecules, the study confirmed that an initial burst release of the protein or the peptide was followed by either a gradual or a steady-state release depending on the cargo. The study was able to demonstrate that the released quantity of the biomolecular cargo can be effectively controlled simply by altering the thickness of the BCP thin film.

#### 3.1.3. BCP Nanodomains for Proteins: Multicomponent Systems

Biomedical applications in many practical settings are expected to involve multiple protein components, rather than single protein species. However, the interaction dynamics and kinetics of multicomponent proteins on solid surfaces are understood much less than single protein component systems in general, let alone for those polymer surfaces of nanoscale topology and chemical variability. Insights from single-component protein studies may not be applicable to adequately explaining more complex, multicomponent protein behaviors on nanoscopic material surfaces. On macroscopic solid surfaces, a protein exchange process known as the Vroman effect has been commonly observed from the competitive interactions of multicomponent proteins. The effect has been extensively documented in the areas of hemostasis, thrombosis, and biomaterials [168,169,170,171,172,173,174]. The Vroman process describes a phenomenon in which proteins, preferentially bound on a solid surface at early times, are displaced by other proteins in the bulk solution over time. Fast-diffusing protein species of lower molecular weights with lower surface affinity tend to arrive at the solid surface at earlier times. These species are replaced later in time by other slow-diffusing protein species of higher molecular weights and higher surface affinity.

Unlike the cases for macroscopic surfaces, not much insight into protein behaviors on nanoscale surfaces can be currently drawn from the literature. There is currently a lack of definitive experimental data at the single biomolecule level for unambiguously revealing multiprotein protein interaction processes on nanoscale surfaces. Despite this, it is crucial to determine the exact molecular mechanism underlying competitive protein–surface interactions on nanoscale surfaces and to reveal the precise compositions of adsorbed proteins at a given time. Furthermore, it is imperative to acquire such experimental evidence and move beyond the present stage of the field where existing postulations deduced from ensemble-averaged measurements are used to speculate on possible kinetics and mechanisms. Considering all these situations, there still is plenty of room to explore competitive protein interactions on nanoscale polymer templates, particularly those attributes examined at the individual protein level. Research efforts have begun to be put forward for the multicomponent protein systems on nanoscale BCP surfaces, leading to important discoveries on protein behaviors that are exclusive to those interaction mechanisms and dynamics at the nanoscopic interfaces.

**Figure 8:**
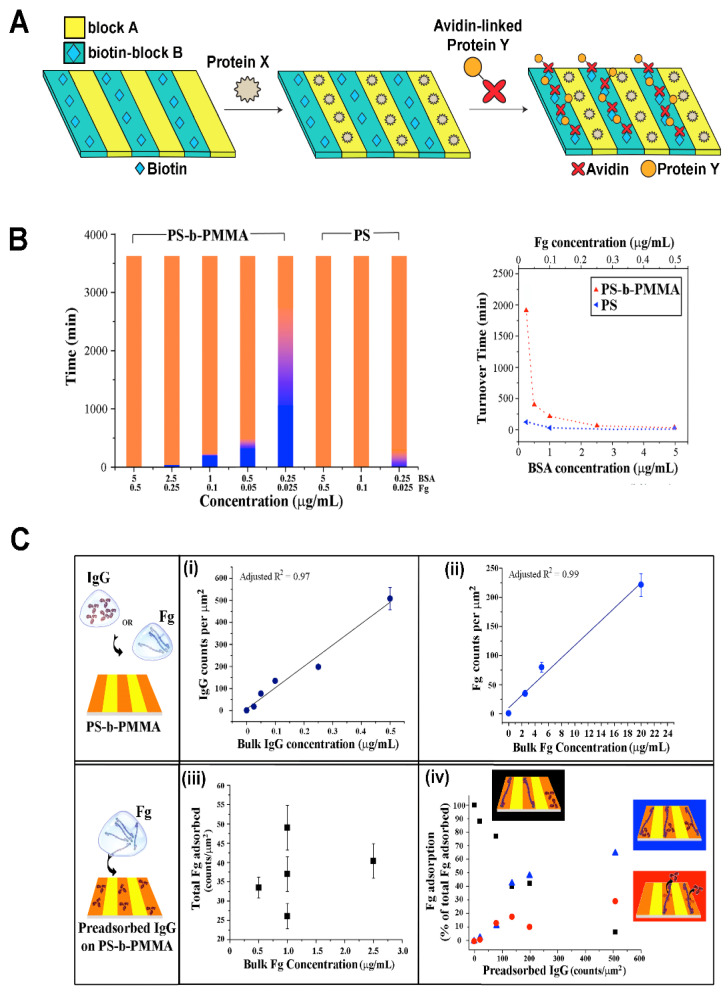
(**A**) The schematic illustration displays an experimental approach that can be used for the spatial control of multicomponent proteins onto self-assembled nanopatterns of an AB-type BCP. The BCP surface contains nanoscale patches of alternating blocks of A and B, where A is more hydrophobic than B and B is pre-functionalized with biotin. Protein X, exhibiting a preferential interaction with the more hydrophobic block A, is first deposited to coat the block A nanodomains. Subsequently, protein Y conjugated with avidin is deposited into the nanodomain areas of block B by way of biotin–avidin interactions. (**B**) Time-dependent behaviors of multicomponent proteins were examined for simultaneous competitive adsorption. The model system involved BSA and Fg simultaneously exposed to the surface of PS-b-PMMA as well as to that of PS homopolymer. At earlier times, BSA was the dominant protein species assembled on the BCP surface. Over time, Fg replaced the BSA molecules on the surface and became dominant. The colored bar graphs display the time-dependent transition between BSA (blue shaded portion) and Fg (orange shaded portion) on the PS-b-PMMA surface as well as on the PS homopolymer template. The different transition stages of BSA-dominant phase, the Fg onset/turnover phase, and the Fg-dominant phase are identified in blue, gradient purple, and orange, respectively. The plot shown in the right panel displays the times corresponding to the turnover point from BSA to Fg for different protein concentrations. Adapted with permission from Ref. [87] Copyright (2016) Royal Society of Chemistry. (**C**) Sequentially occurring, competitive protein behaviors were examined by using the model protein system of IgG and Fg on the surface of PS-b-PMMA. The control data in (**i**,**ii**) were obtained by examining the case of (**i**) IgG and (**ii**) Fg adsorption onto a clean BCP substrate with no preadsorbed proteins. In both cases, the plots show the adsorbed protein amount is linearly dependent on the bulk protein concentration. The data in (**iii**,**iv**) correspond to the BCP surface containing preadsorbed IgG proteins from a prior incubation step. Fg molecules were introduced as a subsequent-stage adsorber. The plot of adsorbed Fg versus Fg bulk concentration in (**iii**) shows that the adsorbed Fg amount was no longer dependent on the bulk Fg concentration. Fg adsorption in this case was dependent on preadsorbed IgG amounts on the surface. The plot in (**iv**) shows the occurrence frequencies of Fg on the BCP surface for the case of distal Fg adsorption (black), proximal Fg adsorption (blue), and Fg replacing IgG (red). Adapted with permission from Ref. [78] Copyright (2018) Royal Society of Chemistry.

**Multicomponent Protein Assembly on BCPs.** Nanostructures formed from a PS-b-PEO derivative, P(S-co-BrS)-b-PEO, have been used for the fabrication of multicomponent biomolecular arrays by combining nonspecific and site-specific interactions between proteins and the BCP nanodomains [80]. The BCP of P(S-co-BrS)-b-PEO contained 5 wt% of 4-bromostyrene (BrS) copolymerized within the PS block for crosslinking with 254 nm light. The PEO segment of the BCP was biotinylated. Various nanopatterns of lines and dots with parallel or perpendicular PEO cylinders with respect to the substrate were generated by using combinations of preparation protocols such as solvent annealing and shadow-mask irradiation. The PS nanodomain areas of the BCP surface were passivated by BSA in order to prevent nonspecific protein adsorption. The biotinylated PEO areas then served as a modular template to pattern neutravidin and biotinylated IgG. The process relied on the specific interaction between biotin and neutravidin to form a complex of biotinylated IgG-neutravidin-biotinylated PEO. A general approach that can be similarly used to pattern multicomponent proteins to the different nanodomain areas of a BCP surface is schematically depicted in Figure 8A.

In a different study, multicomponent protein interactions on PS-b-PMMA were examined for the situation of a simultaneous, rather than sequential, exposure to BSA and Fg [87]. When the protein mixture was applied to the PS-b-PMMA surface, the protein components found on the PS nanodomains of the BCP surface were revealed to be time-dependent. At earlier times, BSA constituted the dominant protein species assembled on the BCP, whereas Fg molecules became the major protein kind that occupied the BCP surface at later times. The data shown in Figure 8B present the change in the dominant protein species on the PS-b-PMMA thin film over time.

**Multicomponent Protein Dynamics on BCPs.** Experimental and simulation research endeavors have been undertaken jointly to reveal competitive protein adsorption behaviors on nanoscale BCP surfaces. When the BCP platform of PS-b-PMMA was exposed to proteins of different kinds such as BSA, Fg, and IgG, it was confirmed that a protein exchange process similar to those on macroscopic polymer surfaces indeed occurred on the nanoscale BCP surface as well [78,87]. However, protein adsorption occurred exclusively on the PS nanodomains regardless of deposition time. Furthermore, the extent to which the initially bound BSA resists its displacement by Fg was much greater on the nanoscale, chemically varying BCP surface relative to the macroscopic, chemically homogeneous surface of PS homopolymer. This phenomenon can be clearly seen in the data presented in Figure 8B. The protein exchange of BSA by Fg took place much more slowly on the nanoscale BCP relative to the PS homopolymer surface [87]. The results indicated that nanoscale BCP surfaces present a more energetically favorable environment for surface-bound proteins which, in turn, enables prolonged residence time of the initially bound protein species and significant retardation in the onset of the protein exchange process.

In another study, individual protein tracking was successfully carried out for competitive protein adsorption of IgG and Fg that occurred in a sequential manner on PS-b-PMMA [78]. The study was able to provide valuable experimental evidence for the dominant adsorption pathway, occurrence frequency, and directionality in protein exchange, all resolved at the single biomolecule level. In addition, the adsorption profiles of subsequent-stage proteins were proven to be significantly different between those sample surfaces with and without pre-adsorbed proteins from earlier stages. For single-component protein adsorption to a neat PS-b-PMMA surface, the protein amount adsorbed on the BCP surface increased linearly with the bulk protein concentration. However, for the sequential interaction case involving a subsequent-stage protein of Fg introduced to the BCP surface treated with IgG in an earlier step, such a linear relationship was no longer observed. In this case, the adsorbed amount of Fg, the subsequent-stage protein, had no dependence on the Fg solution concentration. Rather, the adsorbed amount of the subsequent-stage protein showed a strong correlation to the amount of the prior-stage protein on the BCP surface. The data shown in Figure 8C summarize these features that are associated with the BCP surface under different, competitive adsorption stages in a sequential deposition scenario.

#### 3.1.4. Protein Functionality on BCP Thin Films

Proteins immobilized on a solid surface may present biological functionalities different from those in their native states. The presence of an underlying surface may restrict necessary changes in protein conformation and protein chain rearrangement for exposing its binding sites toward a ligand molecule, for instance. In fact, conflicting results are found in the literature in terms of protein functionality upon surface immobilization. Some reported reduced activities due to substrate-induced, steric hindrance of protein binding to ligands [79,103]. On the other hand, some reported increased protein activity on a solid platform [175,176]. The disparity can be largely explained by the fact that the former conclusion was drawn for protein systems that were randomly adsorbed onto a surface, whereas the latter case involved protein molecules specifically oriented in space with respect to the surface. Tethering of proteins to the platform in the latter case was typically attained by chemical or biological moieties. For protein reactions in solution that occur without the involvement of a solid surface, Brownian motion related to the stochastic chances of biomolecular collisions dominates the reaction process. On the contrary, biomolecules strategically oriented on a surface can guide more effective ligand binding along a well-defined molecular coordinate and increase protein activity. Nevertheless, very little is yet known about the activity and stability of proteins upon their binding onto BCP nanotemplates. It is important to determine the influence of the nanoscale BCP surface on the biofunctionality as well as the stability of proteins for a diverse system of BCPs and biomolecular reactions.

**Protein Activity and Stability on BCP Thin Films.** Research endeavors have begun in this regard using an antigen–antibody system on PS-b-PMMA. Antibody binding activities were examined for IgG molecules bound to a PS-b-PMMA surface using an IgG antibody as well as other control proteins with no specificity to IgG [79]. It turned out that the specificity of IgG molecules in antibody recognition remained on the PS-b-PMMA surface. The IgG molecules on the PS-b-PMMA formed paired complexes only when they were reacted with the IgG antibody, but not in control reactions with nonbinding proteins. In a different study, it was reported that the total enzymatic activity and long-term stability of HRP was greater on a nanoporous BCP thin film when compared to those on the macroscopic surfaces of glass and PS [104]. The nanoporous thin film used in the study was fabricated from a mixture of two BCPs, 90 wt% PS-b-PLLA and 10 wt% PS-b-PEO. The nanoporous BCP platforms provided a greater surface area and easier mass-transfer than the control surfaces. This promoted the enzymatic reactions and increased the catalytic activity of HRP. In another study, biological activities in antibody binding were tested for the proteins of Fg, Mb, and LZM immobilized on a PS-b-PHEMA surface [89]. The immunoreactions were carried out on PS-b-PHEMA by using specific antigen–antibody pairs for each protein, i.e., Fg with anti-Fg, Mb with anti-Mb, and LZM with anti-LZM. It was demonstrated that the amounts of adsorbed antibodies were in qualitative agreement with the number density of the protein molecules that were preassembled on the BCP surface.

In addition to the qualitative assessments, quantitative comparisons of enzyme activities have been made for the case of surface-immobilization versus free solution [79,85,103,140]. Enzymatic activities were quantitatively determined for HRP and tyrosinase molecules that were configured to be BCP surface-bound versus freely moving in a solution. It was revealed that, when compared to the same number of HRP molecules in solution, the enzyme molecules bound to the surfaces of PS-b-PMMA and PS-b-P4VP were able to retain approximately 85% and 78% of the free-state activity, respectively [79,103,140]. The HRP molecules on the BCP templates remained stable and catalytically active even after 100 days, when kept at 4 °C. Other biological activities of BCP surface-bound proteins have also been examined. For example, Fg molecules immobilized on PS-b-PMMA were evaluated for their biofunctionality in the activation of microglial cells [85]. It was shown that the surface-bound Fg retained its cell-activating functionality on the BCP template. The outcomes summarized in this section provide encouraging early data of high protein activity and stability upon immobilization to BCP surfaces. These results suggest that protein nanopatterns assembled with the guidance of BCPs can be exploited to fabricate biofunctional constructs for applications in biosensors and biomaterials.

### 3.2. Biomineral Nanocrystals

The structural anisotropy in various mineralized tissues such as nacre, bone, and dental enamel plays a vital role in their remarkable functionalities [177,178,179]. For example, the high mechanical properties and chemical stability of enamel are due to the intricate spatial organization of hydroxyapatite (HAP) nanocrystals that are bundled to form thick prisms and interprismatic regions of different orientations [180,181]. As the supramolecular organization of matrix proteins in mineralizing tissues largely regulates the nucleation and growth of minerals, various strategies have been explored to create mineralizing material platforms, especially those based on organic matrices. Thin films of BCPs can offer excellent chemical contrasts of nanoscopic dimensions that can be easily varied by altering the size and confinement direction of the nanodomains. BCPs can further be formulated to assemble into 3D scaffolds, even making the incorporation of 3D printing possible to fabricate tailored biomaterials [182]. Therefore, the BCPs’ capability to produce well-controlled 2D and 3D nanopatterns in a facile and rapid manner can present distinctive advantages to the biomineralization field. For instance, protein nanopatterns and peptide nanoassemblies on BCP surfaces can be used to seed mineral filaments and platelets similar to those processes seen in natural biominerals, and further direct a mineralization process with high fidelity. As discussed, spatial control is one of the most critical factors in mineralization since the specific organization of individual nanocrystals and their larger-scale arrangements determine the resulting material’s properties. To this end, BCPs can provide exquisite spatial control at the nanometer scale in guiding mineralization.

**Figure 9:**
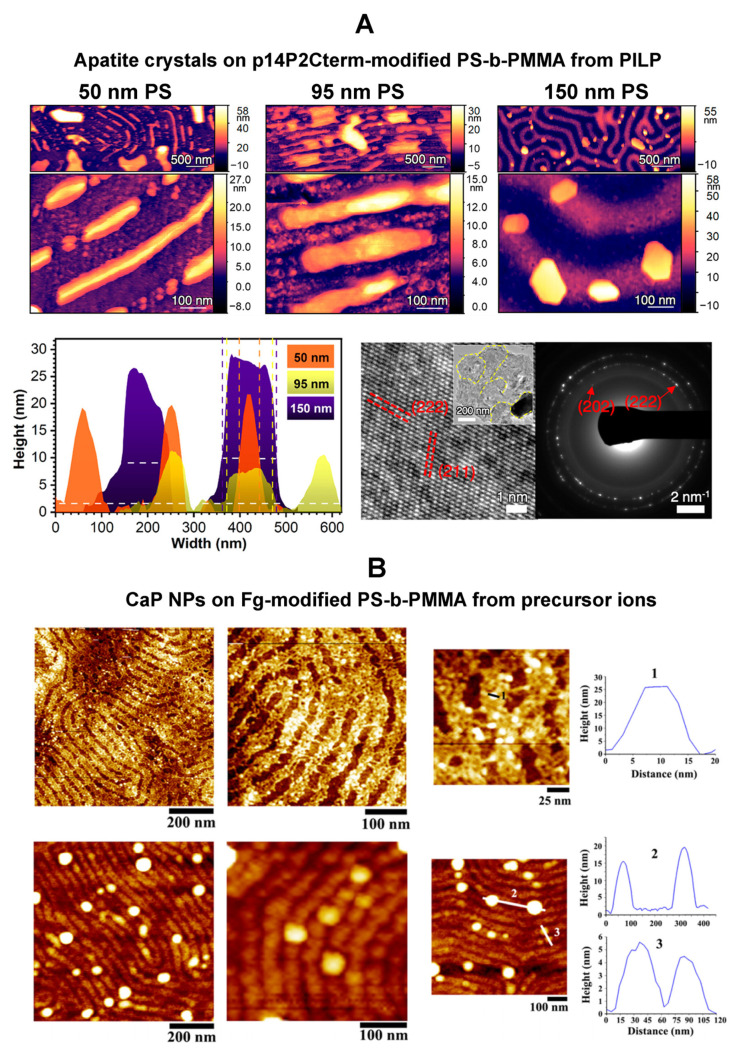
(**A**) The AFM and TEM data were obtained from apatite crystals formed on p14P2Cterm-modified PS stripes of PS-b-PMMA thin films using PILP. The height versus width plot displays the apatite crystal dimensions formed on the BCP templates with 50 nm, 95 nm, and 150 nm p14P2Cterm-PS stripes. White horizontal dashes indicate the base of the particle (PS stripe) and color-coded vertical dashes indicate the filament width. The high-resolution TEM (HRTEM) and selected area electron diffraction (SAED) data were obtained from the particles extracted from the template of 150 nm PS stripe. The TEM data confirmed that the particles were crystalline with lattice and reflections specific to apatite. The inset displays a low-magnification TEM image and corresponding SAED pattern of aggregated single crystals and their grain boundaries as yellow dashed lines. Adapted with permission from Ref. [83] Copyright (2023) American Chemical Society. (**B**) The AFM panels show CaP NPs preferentially nucleated and grown on the Fg-covered PS nanodomains of PS-b-PMMA after (top row) 5 min and (bottom row) 7 h of incubation time in a precursor solution. The particle sizes measured along the white lines are provided for the two different incubation periods in the line analysis panels. Adapted with permission from Ref. [85] Copyright (2016) American Chemical Society.

**Calcium Phosphate Nanoparticle Assembly on BCP Thin Films.** PS-b-PMMA nanopatterns predecorated with a protein layer have been successfully employed to seed calcium phosphate (CaP) NPs [83,85]. CaP-based materials such as HAP and triple calcium phosphate (TCP) are biomedically important materials that are often used to coat the surface of implant biomaterials. The incorporation of the CaP-based materials increases the biocompatibility of implant materials and accelerates the man-made material’s integration with living tissues [183,184]. Aligned PS-b-PMMA has been employed to guide CaP growth after the BCP surface was first patterned by using Amel-derived peptide NRs associated with tooth enamel formation [83]. In the study, two prototypical Amel peptide sequences of p14P2 and p14P2Cterm were used. The peptide NRs bound to the PS domains were able to retain their β-sheet structure and biological activity on the surface and direct the formation of filamentous and plate-shaped minerals of CaP. CaP crystals were mineralized from both an aqueous solution of precursor ions and a polymer-induced liquid-like precursor (PILP). Each mineral was revealed to be a single crystal whose crystalline planes were similar to those of apatite filaments in enamel. By employing nanopattern dimensions ranging from 50 to 150 nm in PS width, it was determined that the width of the apatite crystals was directly dependent on the width of the PS stripes. These results are displayed in Figure 9A. In a different study, nanostriped domains of PS-b-PMMA were used to produce CaP NPs on Fg-covered PS nanodomains. The nanodomain width and orientation were tuned for the alignment of packed Fg molecules in an end-to-end manner parallel to the stripe direction [85]. The BCP template with a densely packed Fg layer was then exploited for nucleating CaP NPs whose results are summarized in Figure 9B. These research efforts have demonstrated that the BCP-directed approach may serve as a highly generalizable platform for nanopatterning of mineral crystals by being able to effectively control the size, number density, and spatial locations of the mineral particles via the underlying BCP nanostructures.

### 3.3. Cell Adhesive Molecules

It is well-understood that proteins organized on macroscale polymer surfaces can function as a cell-mediating layer and ultimately affect cell behaviors of adhesion, proliferation, and differentiation by influencing upstream cytoskeletal dynamics and downstream gene expression. It is also known that the number, morphology, and alignment of cells are affected by the surface density and gradient of cell-corresponding adhesion molecules [185,186,187,188]. The initial cell–surface interactions can be regulated by influencing the presence of these cell-corresponding adhesion molecules on a material surface to which cell receptors bind afterwards. The adhesion molecules usually consist of specific peptide sequences or proteins such as RGD-containing epitopes, FN, collagen (Col), and gelatin. The activation of specific transmembrane receptors such as integrins further induces the assembly of adhesion sites known as focal adhesions [189]. Hence, upon initial cell attachment, cell behaviors can be additionally controlled by altering the degree of integrin binding and focal adhesion formation. This is usually achieved by providing different micro- and nano-environments of chemical cues to a material surface in the form of a protein layer. Proteins such as cytokines, growth factors, hormones, and adhesion molecules are used for this purpose [190]. Chemical cues can also take the form of hydrophobic, Coulombic, and van der Waals forces as well as surface energies between the cell membranes and the underlying polymer surfaces [190].

**BCP Thin Films for Cell Adhesive Molecules.** BCP-based approaches are highly conducive to generating molecular patterns to control the clustering of cell adhesion receptors and structural signaling activities of cell adhesion. This is because protein layers on BCP templates can be exploited as well-defined chemical cues. The spatial organization of protein layers can be achieved spontaneously and instantaneously at nanoscale precision on BCPs for tuning the chemical specificity of adhesive epitopes. In addition, BCP-based methods can be beneficial in regulating the physical features of an epitope-containing platform through adjusting its geometry, rigidity, and spacing. Transplanted cells can recognize and respond to these different nanoscale cues on BCP surfaces in a highly sensitive manner, ultimately affecting the degree of gene expression and tissue formation. Hence, self-assembled BCP nanopatterns show great potential to be utilized for modulating a variety of experimental parameters critical for cell receptor-initiated processes.

BCP surfaces have been engineered to match the bioligand spacing found in cells. BCP templates have also been constructed to provide a nanoscale gradient with varying bioligand spacing in order to monitor changes in cell sensitivity with respect to the spatial distribution of the adhesion ligands. For example, BCP nanotemplates were generated to provide periodic surface sites of ~8 nm in size to match the diameter of integrin in the cell membrane [106]. Nanoscopic topological features were also varied to control the spacing and density of biorecognition molecules for cell receptors which led to substantial changes in cell behaviors [105,106,112]. In one study, a BCP of PS-b-PI with ring-like FN nanopatterns was shown to increase the percent surface coverage and density of Chinese hamster ovary (CHO) cells relative to control substrates [92]. The controls were composed of either a homogeneous FN surface or a PS-b-PI template with striped nanopatterns of FN. It was also found that the PS-b-PI surface with ring-like FN nanopatterns induced more actin fibers, cell spreading, and focal adhesion formation. These results were attributed to a high local FN density on the ring areas, consequently leading to increased integrin clustering and stable focal adhesions.

In another study, an optimal range for the BCP template spacing that is necessary for integrin adhesion and focal adhesion was determined [106]. A BCP surface of PS-b-P2VP was first prepared to produce hexagonally arranged Au dots on each BCP micelle to which c(-RGDfK-) peptides were linked via thiol–Au interactions. When the separation distance between each micelle was greater than 73 nm, cell attachment and spreading as well as the formation of focal adhesions were revealed to be highly restricted. These templates prevented integrin clustering, whose step is important not only for the initial binding of cells to the surface but also for the subsequent cell attachment via stable adhesion sites. The optimal spacing between each micelle was determined as 58–73 nm. This range was considered universal after examining different cells of MC3T3-osteoblasts, B16-melanocytes, REF52-fibroblasts, and 3T3-fibroblasts.

Other BCPs have been employed for nanopatterning RGD peptides as well. For instance, BCP brush samples of polyacrylamide/bis-acrylamide-block-poly(acrylic acid) (PAAm/bisAAm-b-PAA) were prepared with various cross-linking density [101]. The PAA segment of the BCP was then conjugated with an RGD peptide of GRGDS via NHS/EDC chemistry for cell adsorption and spreading. In a different study, the size and spacing of the PEO nanodomains on PS-b-PEO templates were varied as 8–14 nm and 62–44 nm, respectively [91]. These templates were further modified with RGD binding peptides and used for the adhesion of NIH-3T3 fibroblasts. The study identified that the spacing between the PEO nanodomains was crucial for controlling cell spreading on the BCP surfaces. A decrease in the patch spacing for the RGD binding peptides led to an increase in the spreading of NIH-3T3 cells. This approach was later extended to the production of porous 3D PS-b-PEO scaffolds [100]. In this case, PEO nanodomains present throughout the highly porous 3D scaffolds were functionalized with RGD peptides. The 3D presentation of the RGD peptides, correlated directly to the nanodomain structures formed in the original BCP scaffold, was controlled by adjusting the molecular weight of the PS-b-PEO copolymer.

The effect of a chemical cue on cell behaviors due to the variations in the hydrophobic and hydrophilic patches on BCPs has been examined as well [93]. The BCP surface used for this investigation consisted of PMPC and poly(3-methacryloyloxy propyltris(trimethylsilyloxy) silane) (PMPTSSi). Different arrangements of nanoscopic polymer patches were obtained by BCP’s phase reversal processes between the two blocks. The BCP surfaces were then used to produce cell-adhesive nanopatterns of FN molecules on the PMPTSSi patches for subsequent L929 cell adsorption. The BCP micellar geometry of the hydrophilic PMPC core and hydrophobic PMPTSSi matrix led to more adsorption of L929 cells than the opposite block arrangements for the core and matrix components [93].

### 3.4. Cells

Cell interaction with a material surface can steer various biological processes by playing an essential role in the regulation of cell viability, proliferation, and differentiation. Cell adhesion to the surfaces of artificial hearts and hollow dialysis fibers can cause undesirable outcomes such as platelet adhesion and thrombosis [174]. In contrast, promoting cell attachment to artificial scaffolds is critical in cell-based bioarrays and biosensors as well as in tissue engineering and regenerative medicine [190,191]. In some applications, both properties may be simultaneously needed. For instance, materials capable of promoting stem cells and, at the same time, inhibiting cancer cells on platform surfaces are desirable in bone regeneration after injury or pathology. As such, controlling polymer surfaces to facilitate or resist cell interactions to meet specific demands is an important consideration in devising biointerfaces [1]. There exists a great deal of fundamental knowledge and design principles to control cell–material interactions. For instance, it is generally understood that water wettability of a polymer surface is one of the key factors to determine cell behaviors. Protein adsorption, required for subsequent cell activities on the material surface, is known to be largely controlled by the water wettability of the material. However, most of the current knowledge set is based on cell interactions with macroscopic surfaces which may not be adequately carried over to explain cells interfacing nanoscopic surface features.

Cell behaviors on nanoscopic BCP patches with different hydrophobicity and hydrophilicity can drastically differ from what can be deduced by the average wettability values of macroscopic, homopolymer counterparts. Predicting cell behaviors as a function of a simple and single, structural or chemical parameter becomes difficult for BCP surfaces. For example, the lack of cell adhesion and cell proliferation behaviors is widely reported on the homogeneously prepared PMPC surface [81,192]. Yet, entirely different cell adhesion profiles were observed from a heterogeneously prepared PMPC polymer surface. A triblock copolymer platform, composed of hydrophilic PMPC and hydrophobic PDMS as A and B segments of the ABA-type BCP, was fabricated to present nanodomains of vertically arranged PDMS cylinders embedded in a PMPC matrix [81]. It was revealed that many L929 fibroblast cells adhered to the heterogeneously prepared, hydrophilic polymer surface with a water contact angle of less than 20°, even though the hydrophilic monomer composition in the heterogeneously prepared triblock platform was only around 45%. These results suggested that the segregated hydrophobic domains on the BCP platform should be considered for designing polymer-based biomaterials.

Although relatively fewer research attempts have been made for modulating cell behaviors specifically by BCP nanopatterns, there has been a growing interest in exploiting the unique advantages of BCPs to the development of cell-based bioarrays and biomaterials. BCPs can be readily designed to produce nanopatterned surfaces that are stable at physiological conditions, enough to sustain human and other cell viability. BCPs offer adjustable nanomorphology, versatile surface chemistry, and even a possibility for the facile development of non-cytotoxic supports from a plethora of available polymer materials. In addition, the different polymer chemistries, topographical features, and mechanical properties of BCPs can present a unique opportunity for their use as triggers or modifiers of biochemical and biophysical cues that are important to modulating cell behaviors. These aspects are highly beneficial in designing biocompatible materials for implant devices and tissue engineering applications. However, the typical size scale associated with cells, unlike those of individual proteins, are tens of micrometers or larger. As such, the size incompatibility between the characteristic dimensions of cells versus BCP nanopatterns can seemingly pose difficulties in correlating cell behaviors with any nanoscopic variations in the topological or chemical features of the underlying BCPs. Regardless, various research efforts have shown a promising sign that BCPs can be used to efficiently guide and modulate cell behaviors.

**Figure 10:**
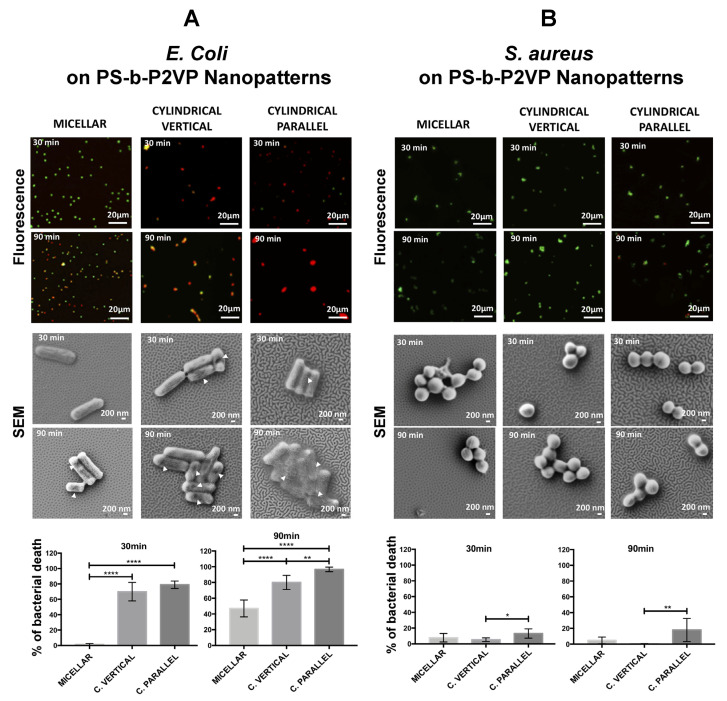
Various nanopatterned surfaces of PS-b-P2VP were used to assess the morphology and viability of (**A**) *E. coli* and (**B**) *S. aureus* cells. They included micellar (~40 nm in thickness, hexagonal micelles of 118 ± 27 nm in diameter), cylindrical vertical (~20 nm in thickness, PS domain of 61 ± 15 nm in width and P2VP domain of 116 ± 34 nm in width), and cylindrical parallel (~40 nm in thickness, PS domain of 62 ± 12 nm in width and P2VP domain of 70 ± 10 nm in width). (**A**) The bacterial cell viability of *E. coli* on the three PS-b-P2VP surfaces was analyzed by SYTO9 (green)/propidium iodide (red) method. SYTO9 was used to visualize those cells with intact and damaged membranes, while propidium iodide targeted only those cells with compromised membranes. When both were present, propidium iodide with a stronger affinity for the cell DNA displaced SYTO9 which, in turn, led to a decrease in green fluorescence inside the cells. White arrows mark damaged bacterial cell walls. Statistical differences: ** *p* < 0.01, **** *p* < 0.0001. (**B**) The fluorescence panels display the cell viability of *S. aureus* after live(green)/dead(red) assays, whereas the SEM panels show the cell morphology on the different PS-b-P2VP nanopatterns. Statistical differences: * *p* < 0.05, ** *p* < 0.01. Adapted with permission from Ref. [109] Copyright (2020) Elsevier.

**BCP Thin Films for Cells.** Studies have reported that different micro- and nano-environments of BCPs can significantly change cellular behaviors by affecting the chemical, topographical, and mechanical nature of cell interactions. PS-b-P2VP nanopatterns were used as-formed without cell adhesion ligands. The effect of various solvent-annealed nanotopographies of the BCP on bactericidal efficiency as well as on cytotoxicity to mammalian cells were subsequently examined [109]. The morphology and viability of *Escherichia coli* (*E. coli*) as well as *Staphylococcus aureus* (*S. aureus*) cells were tested on various PS-b-P2VP templates whose results are shown in Figure 10. It was reported that the cylindrical templates with both the PS and P2VP blocks exposed to the surface exhibited a stronger bactericidal effect than the micellar templates with only PS exposed at the surface. The study also confirmed that the BCP nanopatterns were nontoxic to mammalian cells, making them an efficient platform to resist bacteria.

**Figure 11:**
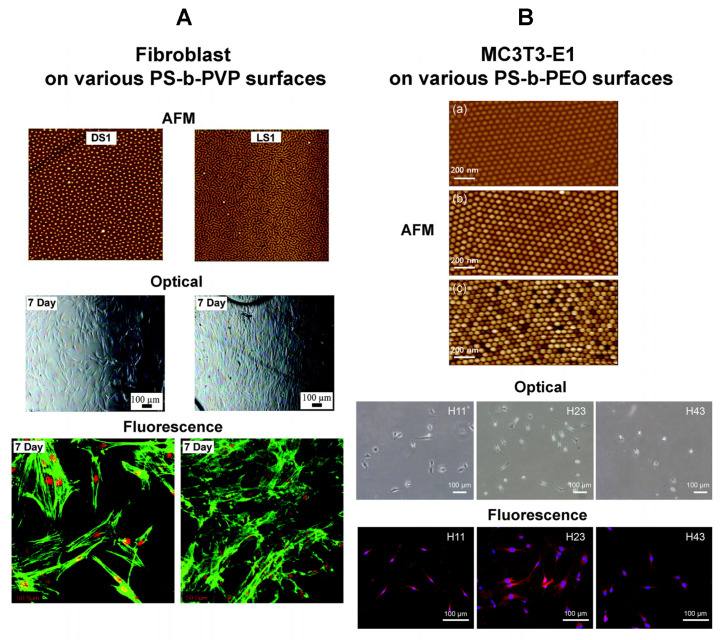
(**A**) Fibroblast cells cultured on two different PS-b-PVP templates of DS1 and LS1 were characterized at day 7 by optical microscopy and confocal fluorescence microscopy. The blank BCP templates before the cell culture are displayed in the AFM images (10 × 10 μm^2^ in size). The DS1 template was prepared from PS_1350_-b-P2VP_400_ to exhibit dot-like nanopatterns of average 200 ± 10 nm in diameter, whereas the LS1 template from PS_610_-b-P4VP_130_ presented lamellar nanopatterns with an average width of 160 ± 7 nm. The confocal fluorescence images show cells stained with phalloidin (actin filaments, green) and propidium iodide (nucleus, red). When compared to fibroblast cells on DS1, earlier cell adhesion and enhanced proliferation were observed from the cells on the LS1 template. Adapted with permission from Ref. [111] Copyright (2007) American Chemical Society. (**B**) MC3T3-E1 cells cultured on various PS-b-PEO/DBSA templates were characterized by optical microscopy and confocal fluorescence microscopy. The AFM panels display PS-b-PEO templates with PS nanopost heights of (**a**) 11 nm, (**b**) 23 nm, and (**c**) 43 nm. Bright areas correspond to PS domains and dark areas correspond to PEO domains. The average diameter of the PS nanoposts and the average center-to-center distance between PS nanoposts were kept constant as 54 nm and 71 nm, respectively, for all templates. The optical and confocal fluorescence images were captured 6 h post-seeding. The three PS-b-PEO/DBSA templates of different PS heights are marked as H11, H23, and H43. The red and blue contrasts in the fluorescence panels are due to vinculin and DAPI, respectively. Cells seeded onto a PS-b-PEO/DBSA surface with 23 nm-high nanoposts showed a much more stretched morphology. Cell growth rate and proliferation increased as the PS nanopost height increased from 11 nm to 23 nm, and then decreased with a further increase in the nanopost height. Adapted with permission from Ref. [112] Copyright (2014) Elsevier.

In a different work, self-assembled nanopatterns of PS-b-P2VP with varying molecular weights and solvent vapor treatments were used to investigate cell morphology and cell adhesion related to bone healing [110]. Distinct cell responses were observed from two osteo-related cell types depending on the topography and chemistry of the BCP nanopatterns. Micellar nanopatterns assembled from a high molecular weight PS-b-P2VP (PS_320_-b-P2VP_398_) promoted the adhesion and spreading of human bone marrow mesenchymal stem cells (BMMSC), whereas the same surface exhibited an opposite effect on osteosarcoma cell line (SaOS-2). In another study, various nanotemplates of heights less than 10 nm were obtained from PS-b-P2VP as well as PS-b-P4VP, and employed for dermal fibroblasts and mesenchymal precursor cells [111]. Different rates of cell adhesion and proliferation were observed on the BCP surfaces, despite the use of BCP substrates with similar surface energies. These differences in cellular response due to the BCP surface-induced nanopatterns are displayed in Figure 11A. It was found that the fibroblasts and mesenchymal precursor cells preferred a BCP template with its nanostructures consisting of a larger nanodomain size and spacing, where the surface of 6 nm in width and 200 nm in spacing was favored over that of 3 nm in width and 160 nm in spacing.

Cell polarization was also examined for MC3T3 osteoblasts on PS-b-P2VP surfaces [105]. The BCP micellar nanolithography used in this study enabled the formation of a quasi-hexagonal, 1–15 nm AuNP array with a tunable NP spacing of 15–250 nm. The AuNPs were biofunctionalized with c(-RGDfK-) in such a way that subsequent binding of integrin molecules to AuNPs followed the stoichiometry of one integrin to one particle. The gradient polarization ratio (GPR) of adherent cells was then analyzed in terms of the axial ratio of the most extended cell width along the direction parallel over perpendicular to the gradient direction on the BCP template. It was found that the cell morphology changed from a radial to elongated shape when the ligand patch spacing was changed from 50 nm to 80 nm. The strongest polarization of cell bodies occurred when the patch spacing ranged between 60 and 70 nm. The study also pointed out that the cells were highly sensitive to even a very small change in the spatial presentation of adhesion ligand patches and responded to a difference in patch spacing of ~1 nm [105].

In addition to the lateral nanoscale dimensions, vertical dimensions of BCP nanostructures that are important for cell behaviors were also identified. The role of nanoscale topology in cell adhesion and differentiation was examined on PS-b-PEO/dodecylbenzenesulfonic acid (PS-b-PEO/DBSA) [112]. The height of self-assembled PS nanoposts that functioned as cell-adhesion domains was varied from 11–43 nm, while keeping both the size and spacing of the nanoposts constant as 54 nm and 71 nm, respectively. The adhesion and growth of mouse preosteoblasts (MC3T3-E1) on these substrates were reported to reach the maximum when the nanopost was 23 nm in height. As for the cell differentiation of MC3T3-E1, the gene expression levels of core-binding factor α1 (Cbfa1) and osteocalcin (OCN) were significantly higher on the 23 nm-tall nanopost surface than on other surfaces. Such changes in cell behaviors that were triggered by the BCP nanotemplate height are summarized in Figure 11B.

The effect of the mechanical properties of an underlying surface on cell behaviors has been scrutinized as well. PAAm/bisAAm-b-PAA samples of various cross-linking densities were prepared to exhibit different mechanical properties [101]. The PAA segment of the BCP was then linked to an RGD peptide. Variation of mechanical properties of the initially polymerized PAAm block was achieved by adjusting the concentration of the cross-linker, bisAAm, while maintaining a constant concentration of AAm. The elastic moduli of the resulting sample surfaces ranged between 600 Pa and 3800 Pa. In subsequent cell studies, changes in cell density and spreading morphology were observed both for NIH 3T3 fibroblast and PaTu 8988t pancreatic tumor cells. The BCP architect of stiffer tethers led to more pronounced cell attachment compared to that of soft un-crosslinked tethers. The study concluded that the rigidity of the underlying BCP significantly influenced the cytoskeleton organization and focal adhesion formation.

Furthermore, aligned nanofeatures as well as triblock copolymer surfaces have been demonstrated for modulating cell behaviors. A gradient fibrous scaffold based on a triblock copolymer of polystyrene-block-poly(ethylene-co-butylene)-block-polystyrene (SEBS) was used to controllably guide the adhesion, spreading, and migration direction of endothelial cells (ECs) [113]. In this work, aligned electrospun fibers of SEBS were treated by selective solvent vapor annealing to produce micrometer and nanometer scale roughness on the surface. The resulting scaffolds texturized with graded fibrous structures were able to function as region-specific, topological guidance for the migration, adhesion, and spreading of the ECs. Nanofibrous micelles have also been prepared from BCPs to mimic the filamentous structure of native extracellular matrix (ECM) and subsequently used to regulate cellular response in tissue engineering. To this end, 2D-organized filomicelles of 46 nm in width, 200 μm in length, and 3–13 GPa in Young’s modulus were produced from the BCP complex based on PS-b-PEO [94]. A combination of fabrication procedures such as out-of-equilibrium nanopattern assembly and soft lithography was employed in order to prepare micellar nanostructures that mimic those of collagen fibrils in native ECM. The fibrous nanostructures of PS-b-PEO were then aligned and immobilized onto a glass substrate and subsequently used for culturing NIH-3T3 fibroblasts. The study showed that the degree of cell alignment increased with the area density of micelles, demonstrating the BCP micelles’ ability to topologically regulate the cellular behaviors on the surface. The area, density, and orientation of the ultra-long PS-b-PEO filomicelles played important roles in modulating the extent of cellular alignment and directionality of the fibroblasts.

## 4. Implications of BCP Nanobiotechnology in Biosensing and Biomaterials

### 4.1. Implications in Solid-State Protein Arrays

**Figure 12:**
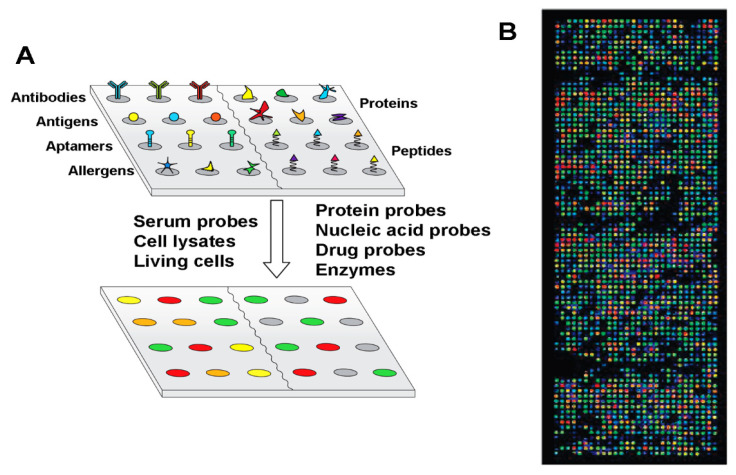
Examples of analytical protein microarrays are displayed. (**A**) Different types of ligands such as antibodies, antigens, DNA or RNA aptamers, carbohydrates or small molecules that are printed onto the surface of the protein array ensure high affinity and specificity to target analytes of interest. The protein chip can be subsequently used for monitoring protein expression levels, protein profiling, and clinical diagnostics. (**B**) Protein microarrays were utilized to carry out multiplexed detection of antibodies in patient sera to tumor antigens. Over 1700 candidate tumor antigens were expressed and captured in a microarray format, and protein expression was detected using anti-glutathione S-transferase (GST) antibody. The arrays were then probed with sera from a healthy individual, and patients with melanoma, breast, and ovarian cancer. Adapted with permission from Ref. [214] Copyright (2008) American Chemical Society.

Solid-state arrays such as protein chips and protein microarrays are widely employed in genomics, proteomics, drug discovery, and clinical diagnostics [4,193,194,195,196,197,198,199,200,201,202,203,204,205,206], as depicted in Figure 12. These microarrays, typically prepared on a microwell or a slide, permit a high degree of multiplexed protein detection from a broad range of sample types including plasma, serum, tissues, and biofluids [194,195,201,202,203,207,208]. One critical aspect of the protein microarray technology is to precisely control the number and spatial distance of biomolecules that are linked to the array surface during its manufacturing stage [197,198,201,209,210,211,212,213]. The surface modification steps to pre-link biomolecules and print proteins on the array surface are necessary to provide specificity in subsequent biorecognition processes that occur between the pre-configured receptor molecules and target analytes. Another critical aspect of microarray technology is to provide stability and functionality of those protein molecules on the array surface. High and long-lasting bioactivity of the surface-bound molecules is a pre-requisite to ensuring successful bioassays and biodetection. The spot size and density in protein arrays are yet another important aspect of microarray technology. The protein spot size and density in conventional protein arrays are typically in the micrometer range. Shrinking these protein patterns down to the nanometer scale will be beneficial to the creation of miniaturized devices and sensors that are capable of low-volume, low-cost, and high-throughput assays. Reduced dimensions in biodevices will also permit minimally invasive detection.

BCP-based schemes can represent a highly versatile and effective approach to rapidly prepare protein patterns into nanometer-sized spots and spacings. The approach relies solely on the self-assembly process of the polymers and that of the proteins. In addition, the chemical compositions of BCPs can be conveniently varied to offer multiple chemical functionalities as needed. Moreover, since the BCP strategy involves a thin film-based approach to produce protein nanopatterns, the resulting constructs can be easily integrated into a flexible sensor. Therefore, the BCP-based methodology is conducive to the recent trend in designing bioplatforms, i.e., miniaturization combined with chemical variety and mechanical flexibility. The advantages of the BCP-guided strategy present great potential for a low-cost, low-reagent volume, multiplexed, and high-throughput bioplatform whose architecture can be small, flexible, and portable. Overall, BCPs can greatly benefit the current field of protein micro-/nano-array technology.

### 4.2. Implications in Quantitative Bioanalyte Detection

The various research endeavors discussed earlier have begun to reveal intriguing behaviors of proteins and cells unique to nanoscale BCP interfaces. The fundamental knowledge obtained, in turn, can serve as guiding principles much needed for the design and application of next-generation nanobioarrays. One of the key findings directly influences the crucial aspect of reliably performing quantitative protein detection. In the BCP-guided assembly of proteins and cell adhesive molecules, it was possible to precisely control the number, surface density, and surface coverage of biomolecules [75,79,85,103,105,106,140,156,158]. The fact that such control in bioquantification can be attained through self-assembly by simply tuning the size of the underlying BCP nanodomain with respect to that of the biomolecule of interest is highly advantageous [37,75,85,140,156]. Another important discovery pertains to those systems whose length scales of the BCP surface features are commensurate with the characteristic dimensions of individual biomolecules [77,85,151,153,156,158]. In these cases, even a relatively small, topological and chemical change of the underlying BCP surface can result in strikingly different behaviors from biomolecules. This effect applies not only to those biomolecules directly interacting with BCP templates but also to those biomolecules indirectly coupled to the templates via prior-stage proteins or other bioligands.

The drastic surface-induced changes can be best evidenced in research efforts that systematically compared the interaction behaviors of the same proteins on BCP nanodomains relative to homopolymers, random copolymers, and polymer blends [35,76,77,78,85,87,156,158]. Salient features such as high protein density and tight 2D-packing represent those behaviors distinctively observed from nanoscale BCP platforms. When comparing the number of protein molecules per given polymer surface footprint, the largest number of proteins was found on nanoscale BCP platforms whose feature sizes were well-matched to that of an individual protein [77,85,151,156]. It was also found that the enhanced protein amount on a BCP surface was not a mere average of those amounts measured on its homopolymer counterparts. In addition, protein molecules on BCPs exhibited a well-ordered, close-packing behavior that resembled an orderly arrangement of atoms in a 2D inorganic crystal. In contrast, the spatial distribution of the same protein molecules did not show any spatial order on the homopolymer counterparts locally or globally.

BCPs’ capacities summarized above can combinedly offer controllability and predictability in fabricating protein nanoarrays. The exact number, density, coverage, and mass of surface-bound protein molecules intended for a particular biodetection can be attained by varying the BCPs’ topological cues (lateral and vertical dimensions, periodicity, and orientation of the nanodomains) with respect to the protein dimensions. They can also be attained by changing the BCPs’ chemical cues (chemical composition, chemical interface, hydrophobicity, and surface charge) for modulating preferential protein–polymer segment interactions. When effectively controlled with these BCP surface-driven factors, the resulting bioarrays will offer a well-defined number of protein molecules organized into nanoscopic spots with a nanoscale periodicity which, in turn, can permit quantitative detection of bioanalytes in a highly reliable manner.

### 4.3. Implications in Stable Biosensors with High Functionality

The stability and activity of biomolecules are both extremely important criteria to consider in bioarray and biosensor applications. Even with a well-controlled number of biomolecules per spot, each of those immobilized biomolecules should be stable and active for the successful quantification of bioanalyte molecules and comparison of detection signals obtained from different spots in a bioarray. As discussed earlier, research efforts have confirmed that high biological stability and functionality are maintained for many different biomolecules assembled on BCP surfaces. Examples of those biomolecules included enzymes, antibodies, serum proteins, peptides, and cell adhesive proteins [76,78,79,85,87,103,105,106,140,158,215]. In comparison to macroscale homopolymer surfaces, biomolecules on nanoscale BCP surfaces are revealed to be much more stable and functional. The higher protein stability of BCPs is attributed to several factors such as the presence of high interfacial density, amphiphilicity, and protein–nanodomain size matching [35,85]. Relative to chemically uniform homopolymers, BCP surfaces can be fabricated to exhibit a high density of chemical interfaces between the nanoscopic domains of chemically distinctive polymer segments. As polymer segments in BCPs show different chemical properties of hydrophilicity/hydrophobicity and surface charge, the outer region of a nanodomain near the chemical interface presents a richer chemical environment than the center region of a nanodomain. As for proteins, their exterior surfaces are quite complex in chemical nature as well. The surface of a protein contains a large number of amino acid moieties with varying charges, polarity, and hydrophilicity/hydrophobicity. Hence, the richer the chemical environments are on a platform surface, the stabler the proteins are on the platform. BCP surfaces satisfy this requirement for protein stability.

**Figure 13:**
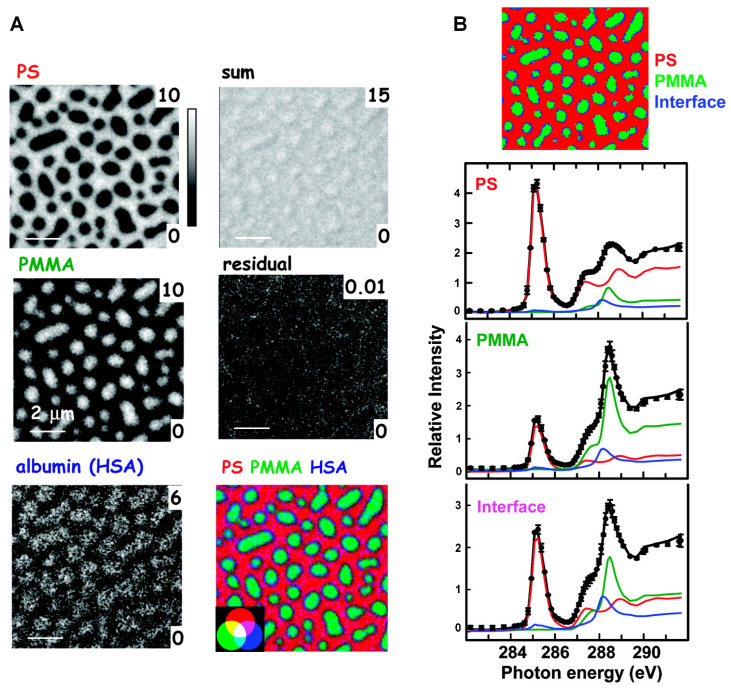
XPEEM component maps are displayed for HSA adsorbed on a PS/PMMA blend film. (**A**) The PS, PMMA, and HSA component maps show the location of each component on the film. The numbers in the upper and lower right of each component map are the minimum and maximum thicknesses in nm for the gray scales. The gray scale for the residual fit is the deviation of the fit and the measured signal, averaged over all photon energies. A composite map is also provided in the last panel where PS, PMMA, and HSA are shown in red, green, and blue, respectively. (**B**) The composite map and the plots for each component are obtained from HSA deposited on the PS/PMMA film using five different pH conditions. PS, PMMA, and HSA signals are shown in red, green, and blue, respectively. All data were derived from C 1s image sequences. Reproduced with permission from Ref. [217] Copyright (2008) American Chemical Society.

In addition, the closer the BCP feature size is to that of a protein, the stabler the protein is on the platform. This is because various amino acid moieties on the exterior surface of the protein can be better stabilized by the amphiphilic environments of the BCP’s interfacial regions [75,77,151,156,157,216,217]. Figure 13 displays synchrotron radiation-based, X-ray photoemission electron microscopy (XPEEM) data which maps the spatial distribution of HSA and the two polymer components of PS and PMMA on a PS/PMMA blend film. The densest layer of HSA was located at the interfaces between PS and PMMA. Similar localization behaviors were also observed from HSA adsorbed on a blend film of PS and polylactide (PLA), where the signals for proteins were highest along the interfacial lines between PS and PLA [216]. This can be also explained by the fact that the nanodomain regions near the chemical interface can stabilize a greater fraction of amino acid residues of the inherently amphiphile protein molecules, thus promoting protein binding at the interfacial as supposed to central sites of the nanodomains. The importance of the amphiphilicity provided by the underlying BCP surfaces is also emphasized in a cell study [81]. Stable cell adhesion was generally promoted on a BCP surface with its hydrophilic domains periodically spaced by hydrophobic domains, whereas a control surface consisting of only the hydrophilic polymer domain was repellant to cell adhesion. Therefore, in addition to the spatial nanopatterning ability to create self-assembled biomolecules, BCPs can provide the structural and chemical environments that are necessary to ensure the stability and functionality of biomolecules upon immobilization to bioarray and biosensor surfaces.

### 4.4. Implications in Tuning Protein Resistance

Being able to tune the protein resistance of a polymer surface is extremely important for the development of biomaterials and biomedical devices. The conventional way to attain low or high protein resistance is by changing the chemical nature of a polymer [218]. The main strategies to attain protein resistance depend on improving surface hydrophilicity through a chemical modification process or incorporation of surface modifying additives. Such surface hydrophilization agents can be incorporated into polymer surfaces either by physisorption, hydrogel network formation, surface grafting, layer-by-layer (LbL) assembly, or additive blending with base polymers [218,219,220]. In some cases, both the anti-biofouling capacity and protein resistance of BCPs are increased by chemically modifying the BCP side chains. For example, BCPs containing fluoroalkyl side chains were produced to form protein-resistant surfaces of polystyrene-block-poly(ethoxylated fluoroalkyl acrylate) (PS-b-PAA–AMP) and poly(hydroxyethylacrylamide)-block-poly(1H,1H-pentafluoropropyl methacrylate) (PHEAA-b-PFMA) [221,222].

It is worthwhile to point out a highly promising but yet-to-be-explored pathway that is different from the conventional routes discussed above. Insights from the aforementioned research endeavors involving BCPs suggest that protein resistance can be effectively tuned by the size scale of polymer surface features, without having to modify their chemical compositions. For instance, the difference in IgG binding behavior to PMMA is intriguing when it is compared between the cases of PMMA homopolymer versus PS-b-PMMA surfaces [156]. As the separation distance between the chemical interfaces of the polymer segments approaches several tens of nm in the BCP template from infinity in the case of PMMA homopolymer, no IgG molecules bind to the PMMA areas on the nanoscale BCP template. This is drastically different than the behavior of the same protein on PMMA homopolymer, where IgG molecules readily bind to PMMA. This outcome suggests that the nature of protein fouling and antifouling behaviors to given polymers can be dramatically changed when a polymer surface of the same chemical composition becomes nanosized and surrounded by chemical interfaces. It further indicates that the tendency of a given polymer to act as a protein-attracting or -repelling platform may be altered by simply tuning the size scale of the polymer interface down to that of an individual protein, instead of changing the chemical composition of the polymer.

### 4.5. Demonstration of Biosensors

**Figure 14:**
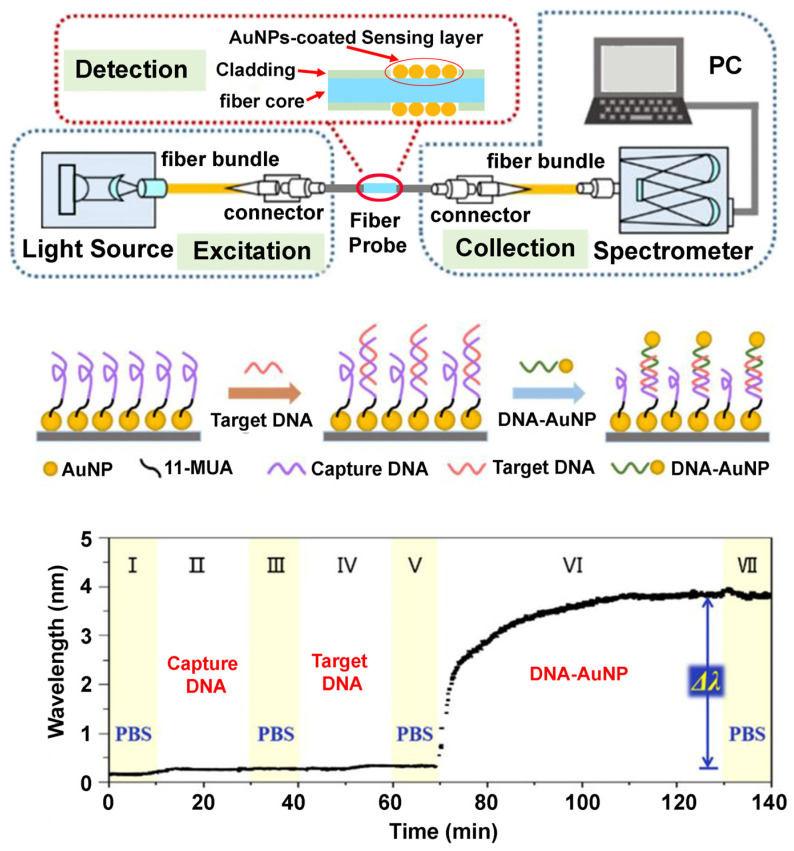
The schematics display an AuNP-based, fiber-optic LSPR sensor using a BCP-templating technique that was employed for the detection of ssDNA hybridization. The plot of wavelength versus time shows the sensor responses for different DNA fragments (capture DNA, target DNA, and DNA-AuNP) as well as for phosphate-buffered solution (PBS) during the hybridization reactions. Adapted with permission from Ref. [114] Copyright (2021) MDPI.

Several examples are found in the literature in which the BCP thin film-based biomolecules were utilized in optical and electrochemical biosensors with specific sensor characteristics such as the detection limit and dynamic range [114,115,116,117]. Most of these studies involve a conventional material surface as a base platform onto which BCP nanostructures were applied for modification with biomolecules. For example, an approach to use AuNPs in conjunction with a BCP has been successfully employed to create a highly sensitive, BCP-templated, optical fiber for DNA detection. Figure 14 displays the overall approach for the fiber optic sensor using AuNPs/BCP. A conventional optical fiber surface was first treated with PS-b-P4VP whose nanopatterns were then used for linking AuNPs to the P4VP nanodomains [114]. Single-stranded DNA (ssDNA) hybridization reactions for *rop* B gene were carried out by using the BCP-templated LSPR sensor, whose surface contained capture ssDNA tethered to AuNPs as signal amplification tags. The employment of AuNPs enabled an optical phenomenon known as localized surface plasmon resonance (LSPR) which, in turn, led to high sensitivity in the detection of *rop* B gene related to Rifampicin-resistant tuberculosis. The study reported a detection limit of 67 pM and a dynamic range of 10^−10^–10^−6^ M for the *rop* B detection.

**Figure 15:**
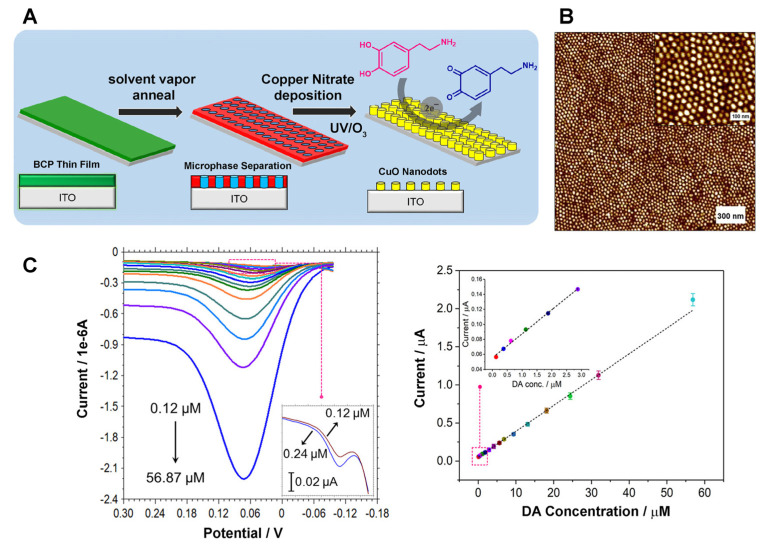
(**A**) The schematic shows the PS-b-P4VP thin film-based sensor fabrication process for developing CuO nanodots on an ITO substrate as well as the mechanism for the sensor operation in detecting DA. (**B**) The AFM image displays CuO nanodots on a Si substrate produced by using the PS-b-P4VP thin film and copper nitrate infiltration process. (**C**) The plots in the left panel correspond to the differential pulse voltammetry (DPV) data of the sensor in the presence of various DA concentrations in PBS pH 7.4. The calibration graph of the sensor for the determination of DA is provided in the right panel. Adapted with permission from Ref. [117] Copyright (2019) American Chemical Society.

BCP thin film-based efforts have been made to develop electrochemical sensors as well. The surface of a conventional Pt electrode was first modified with a film of PS-b-P4VP micelles. The BCP-modified electrode surface was subsequently treated to immobilize glucose oxidase (GOx) for the detection of glucose [115]. The study demonstrated that the BCP surface provided excellent stability for the enzyme molecules. The enzymatic activity and selectivity were maintained on the BCP surface for the glucose detection, resulting in a detection limit of 0.05 μM and a linear range of 10–4500 μM. Similarly, a BCP thin film of poly(n-butylmethacrylate)-block-poly(N,N-dimethylaminoethyl methacrylate) (PnBMA-b-PDMAEMA) micelles was adsorbed to the surface of conductive materials of graphite and Au. Then, choline oxidase (ChO) was immobilized onto the BCP template to examine the enzymatic activity towards choline [116]. The resulting amperometric choline sensor exhibited a detection limit of 30 nM and a linear range of 30 nM–100 μM. In a different study, a BCP thin film of PS-b-P4VP coupled with CuO nanodots was developed into an electrochemical biosensor and subsequently used for sensitive and selective determination of dopamine (DA) [117]. In this work, the BCP nanotemplate was prepared atop an ITO substrate that served as a working electrode. A porous nanotemplate was then generated via solvent vapor annealing of the BCP thin film, after which a copper nitrate solution was spun-coated onto the porous nanostructure. An ensuing treatment of UV/O_3_ yielded CuO nanodots on the film. Figure 15A displays the overall approach used to produce a BCP-based DA sensor with CuO nanodots as an active DA-sensing element. The voltametric behavior of the oxidation reaction of DA on the sensor was examined by using cyclic voltammetry and differential pulse voltammetry. The current signal of the sensor was linear within a DA concentration range of 0.12–56.87 μM, with a sensitivity of 326.91 μA mM^−1^cm^−2^ and a detection limit of 0.03 μM. Figure 15B,C show the analytical characteristics of the DA sensor. The work demonstrated that a BCP-based approach can be applied to create a robust and reliable DA sensor that has a critical importance in neurological disorders.

Overall, biosensing applications that specifically exploit biomolecules on BCP nanopatterns are currently limited. However, the versatility and flexibility of BCPs in meeting the growing demand for miniaturized and flexible sensor arrays can open additional pathways for practical applications. The likelihood for the development of next-generation BCP-based biosensors and biomaterials is even greater when considering a plethora of biomolecules that can be easily self-assembled and guided by BCP nanopatterns. New types of BCP-based biointerfaces such as those enabling stimuli-responsive and time-programmed release of biomolecules may soon be realized as the research field becomes more mature. Continuous efforts in this direction will ultimately provide BCP-based, preventive and therapeutic measures in nanomedicine.

## 5. Outlook and Conclusions

The pioneering research endeavors on BCP–biomolecule nanoassembly and nanoconstructs discussed in this paper present new avenues for building biosensors, biodevices, and biomaterials with exquisite control at the nanoscale level. Many inspiring outcomes have already begun to provide definitive experimental evidence as well as computer simulation predictions on the mechanisms and kinetics of biomolecule–nanoscale surface interactions at the single-molecule level. One of the key messages from previous studies is that the behaviors of biomolecules on nanoscale, chemically rich surfaces are drastically different from those observed on bulk or macroscopic surfaces with no chemical variations. Another important lesson from the earlier efforts is that the interaction behaviors of biomolecules become even more distinct on surfaces that feature structural and chemical variations at the length scale commensurate with single biomolecule dimensions.

Future research engagements are still highly warranted in order to address the fundamental questions that have not yet been answered and further guide the current capacity to rationally design custom-tailored biointerfaces tuned at the nanoscale. Compared to what is currently known for single-component biomolecule behaviors on BCP surfaces, interfacial interactions involving multicomponent biomolecules are far less understood. Much of the dynamic, time-dependent interaction behaviors of biomolecules on BCP surfaces are still unknown. The structure–function relationships should be determined from a wider range of biomolecules on BCP surfaces beyond the present knowledge. Such efforts will provide answers to how structural and chemical variations on BCP surfaces can be used to ultimately modulate the function of the surface-bound biomolecules. Relative to those systems of proteins, cell adhesive molecules and cells, little is understood about the interaction behaviors of nucleic acids such as DNAs on BCP nanopatterns. Unlike cell adhesion, less is known for cell alignment, migration, and differentiation on BCP surfaces. It largely remains unclear the exact mechanisms through which cells sense a very small variation in the topological, mechanical, and chemical on BCP surfaces other than those cases where local directional cues are varied by the density of adhesion molecules. Similarly, how the nanoscale structures of proteins on BCP surfaces affect cell behaviors is unclear.

Looking ahead, plenty of exciting research opportunities await to advance the research field of BCP nanobiotechnology by capitalizing on the fundamental groundwork discussed in this paper. Future research efforts are needed to explicitly demonstrate biomedical applications that are beyond those limited examples currently available at the proof-of-concept level. Future developments in this direction should be geared towards significantly improving their practical applications. For this goal, additional research endeavors are warranted for the development of novel BCP–biomolecule nanoconstructs that are small and noninvasive with built-in chemical and biological functionalities needed in biosensing and biomaterials. In addition, the practical utilities of BCP–biomolecule nanoarrays as miniaturized, high-throughput, and flexible biosensing platforms are still to be proven. Incorporations of BCP–biomolecule nanoconstructs into field-ready, medical implants and medical devices are also to be demonstrated. For these applications, new types of BCP-based biointerfaces are to be designed for controlling protein- and antimicrobial-resistance as well as for time-dependent loading/unloading of cargo molecules into/from the biointerfaces. For cell-based applications, nanotextured BCP biomaterials are yet to be developed and demonstrated for selectively propagating a particular cell type and further optimizing specific cell adhesion and proliferation.

In summary, nanoscale-controlled surface organization of functional biomolecules guided by BCP thin films presents a new paradigm in nanobiotechnology. BCP nanobiotechnology can enable the future development of nanobiosensors and nanobiomaterials with superb spatial and functional control whose fabrication processes are solely driven by the self-assembly of the component elements. New intriguing characteristics of biomolecule interactions have begun to emerge from the previous research efforts highlighted in this paper. These research endeavors have laid the important groundwork for advancing BCP nanobiotechnology. Future research directions should focus on narrowing the current knowledge gap in nanoscale versus macroscale as well as ensemble versus single biomolecule behaviors on polymer surfaces and, also, on promoting the practical utility of BCP–biomolecule nanoconstructs in biosensing, biomaterials, and medical devices.

## Figures and Tables

**Table 1:** Various BCPs and biosystems demonstrated for controlling key characteristics of biomolecules via BCP nanopatterns.

Biomolecule Name (Abbreviation)	BCP Nanotemplate Used with Biomolecules	Section Covered	Ref.
**Proteins and Peptides**			
Immunoglobulin G (IgG)	Polystyrene-block-polymethylmethacrylate (PS-b-PMMA)	3.1.1. 3.1.2. 3.1.3. 3.1.4.	[75] [76,77] [78] [79]
	Poly(styrene-co-4-bromostyrene)-block-polyethylene oxide (P(S-co-BrS)-b-PEO)	3.1.3.	[80]
	Poly(2-methacryloyloxyethyl phosphorylcholine)-block-poly(dimethylsiloxane) (PMPC-b-PDMS)	3.1.1.	[81]
S-layer protein (SbpA)	Polystyrene-block-polyethylene oxide (PS-b-PEO)	3.1.1.&3.1.2.	[82]
	Polystyrene-block-poly(2-vinylpyridine) (PS-b-P2VP)	3.1.2.	[82]
Amelogenin (Amel)	PS-b-PMMA	3.1.1. 3.2.	[83]
Fibrinogen (Fg)	PS-b-PMMA	3.1.1. 3.1.2. 3.1.3. 3.1.4. 3.2.	[84,85] [86] [78,87] [85] [85]
	Polystyrene-block-poly(2-hydroxyethyl methacrylate) (PS-b-PHEMA)	3.1.2. 3.1.4.	[88,89] [89]
γ-globulin	PS-b-PMMA	3.1.2.	[86]
Fibronectin (FN)	PS-b-PMMA	3.1.2.	[86]
	PMPC-b-PDMS	3.1.2.	[81]
	Polymethylmethacrylate-block-polyacrylic acid (PMMA-b-PAA) Polymethylmethacrylate-block-poly(2-hydroxyethyl methacrylate) (PMMA-b-PHEMA) Polyacrylic acid-block-polymethylmethacrylate-block-polyacrylic acid (PAA-b-PMMA-b-PAA) Polymethylmethacrylate-block-poly(2-hydroxyethyl methacrylate)-block-polymethylmethacrylate (PMMA-b-PHEMA-b-PMMA)	3.1.2.	[90]
	PS-b-PEO	3.1.2.	[91]
	Polystyrene-block-polyisoprene (PS-b-PI)	3.3.	[92]
	PMPC-block-poly(3-methacryloyloxy propyltris(trimethylsilyloxy) silane) (PMPTSSi)	3.3.	[93]
Thrombomodulin (TM)	PS-b-PMMA	3.1.2.	[86]
Type I collagen (Col I)	PS-b-PMMA	3.1.2.	[86]
Collagen fibrils	PS-b-PEO	3.4.	[94]
Human/bovine serum albumin (HSA/BSA)	PS-b-PMMA	3.1.3.	[78,87]
	PS-b-PI	3.1.2.	[95]
Ovalbumin (OVA)	Poly(acrylic acid)-block-poly(N-isopropyl acrylamide) (PAA-b-PNIPAM)	3.1.2.	[96]
Streptavidin (SAv)	Polyethylene glycol-block-polystyrene (PEG-b-PS)	3.1.2.	[97]
Myoglobin (Mb)	Polystyrene-block-poly(2-hydroxyethyl methacrylate) (PS-b-PHEMA)	3.1.2.&3.1.4.	[89]
	PS-b-PEO	3.1.2.	[98]
Lysozyme (LZM)	PS-b-PHEMA	3.1.2.&3.1.4.	[89]
	PS-b-PEO	3.1.2.	[99]
Green fluorescent protein (GFP)	PS-b-PEO	3.1.2.	[91]
Arginine-Glycine-Aspartate (RGD) peptide motifs	PS-b-PEO	3.1.2. 3.3.	[91] [91,100]
	Polyacrylamide/bis-acrylamide-block-poly(acrylic acid) (PAAm/bisAAm-b-PAA)	3.3. 3.4.	[101]
TAT peptide	PS-b-PEO	3.1.2.	[99]
Coiled-coil α-helix bundle (heme-binding motif)	PS-b-PEO	3.1.2.	[98]
Lsmα protein	PS-b-PEO	3.1.2.	[102]
Horseradish peroxidase (HRP)	PS-b-PMMA	3.1.4.	[79,103]
	Polystyrene-block-polyethylene oxide/polystyrene-block-poly(l-lactide) (PS-b-PEO/ PS-b-PLLA)	3.1.2. 3.1.4.	[104]
avß3 integrin receptor of c(-RGDfK-)	Polystyrene-block-poly(2-vinylpyridine) (PS-b-P2VP)	3.1.2. 3.3.	[105,106]
Tyrosinase	PS-b-PMMA	3.1.4.	[79]
**Nucleic Acids**			
DNA origami	PS-b-PMMA	3.1.2.	[68,107]
	PS-b-P2VP	3.1.2.	[108]
**Cells**			
Chinese Hamster ovary cells (CHO)	PS-b-PI	3.3.	[92]
MC3T3-osteoblasts	PS-b-P2VP	3.3.	[105,106]
B16-melanocytes	PS-b-P2VP	3.3.	[106]
REF52-fibroblasts	PS-b-P2VP	3.3.	[106]
3T3 and NIH-3T3 fibroblasts	PS-b-P2VP	3.3.	[106]
	PS-b-PEO	3.3. 3.4.	[91,100] [94]
	Polyacrylamide/bis-acrylamide-block-poly(acrylic acid) (PAAm/bisAAm-b-PAA)	3.4.	[101]
L929 fibroblasts	PMPC-block-poly(3-methacryloyloxy propyltris(trimethylsilyloxy) silane) (PMPC-b-PMPTSSi)	3.3.	[93]
	PMPC-b-PDMS-PMPC	3.4.	[81]
*Escherichia coli* (*E.coli*)	PS-b-P2VP	3.4.	[109]
*Staphylococcus aureus* (*S.aureus*)	PS-b-P2VP	3.4.	[109]
Bone marrow mesenchymal stem cells (BMMSC), Mesenchymal precursor cells	PS-b-P2VP	3.4.	[110]
	PS-b-P2VP Polystyrene-block-poly(4-vinylpyridine) (PS-b-P4VP)	3.4.	[111]
Osteosarcoma cells (SaOS-2)	PS-b-P2VP	3.4.	[110]
Dermal fibroblasts	PS-b-P2VP PS-b-P4VP	3.4.	[111]
Mouse preosteoblasts (MC3T3-E1)	Polystyrene-block-poly(ethylene oxide)/dodecylbenzenesulfonic acid (PS-b-PEO/DBSA)	3.4.	[112]
Pancreatic tumor cells, PaTu 8988t	PAAm/bisAAm-b-PAA	3.4.	[101]
Endothelial cells (ECs)	Polystyrene-block-poly(ethylene-co-butylene)-block-polystyrene (SEBS)	3.4.	[113]
**Biomineral Nanocrystals**			
Calcium phosphate (CaP), Hydroxy-apatite (HAP), Triple CaP (TCP)	PS-b-PMMA	3.2.	[83,85]
**Biosensors**			
*rop* B gene	PS-b-P4VP	4.4.&4.5.	[114]
Glucose oxidase (GOx)/Glucose	PS-b-P4VP	4.5.	[115]
Choline oxidase (ChO)/Choline	Poly(n-butylmethacrylate)-block-poly(N,N-dimethylaminoethyl methacrylate) (PnBMA-b-PDMAEMA)	4.5.	[116]
Dopamine (DA)	PS-b-P4VP	4.5.	[117]

## Data Availability

Not applicable.
